# Unraveling the genetics underlying micronutrient signatures of diversity panel present in brown rice through genome–ionome linkages

**DOI:** 10.1111/tpj.16080

**Published:** 2023-01-18

**Authors:** Erstelle A. Pasion, Gopal Misra, Ajay Kohli, Nese Sreenivasulu

**Affiliations:** ^1^ International Rice Research Institute Los Baños Laguna 4030 Philippines

**Keywords:** biofortification, epigenetics, GWAS, gene regulatory network, micronutrients, systems‐genetics

## Abstract

Rice (*Oryza sativa*) is an important staple crop to address the Hidden Hunger problem not only in Asia but also in Africa where rice is fast becoming an important source of calories. The brown rice (whole grain with bran) is known to be more nutritious due to elevated mineral composition. The genetics underlying brown rice ionome (sum total of such mineral composition) remains largely unexplored. Hence, we conducted a comprehensive study to dissect the genetic architecture of the brown rice ionome. We used genome‐wide association studies, gene set analysis, and targeted association analysis for 12 micronutrients in the brown rice grains. A diverse panel of 300 resequenced *indica* accessions, with more than 1.02 million single nucleotide polymorphisms, was used. We identified 109 candidate genes with 5–20% phenotypic variation explained for the 12 micronutrients and identified epistatic interactions with multiple micronutrients. Pooling all candidate genes per micronutrient exhibited phenotypic variation explained values ranging from 11% to almost 40%. The key donor lines with larger concentrations for most of the micronutrients possessed superior alleles, which were absent in the breeding lines. Through gene regulatory networks we identified enriched functional pathways for central regulators that were detected as key candidate genes through genome‐wide association studies. This study provided important insights on the ionome variations in rice, on the genetic basis of the genome–ionome relationships and on the molecular mechanisms underlying micronutrient signatures.

## Significance Statement

A set of 109 candidate genes was identified that contribute to the genetic basis of variation in the content of 12 micronutrients in rice. Superior alleles for critical genes explaining high percentage phenotypic variation explained were missing in the improved elite breeding lines, suggesting the value of our results in improving the micronutrient content of the grains to address the bane of hidden hunger positively.

## INTRODUCTION

Micronutrient malnutrition also known as “Hidden Hunger” affects 2 billion people globally (Khush et al., 2012). Micronutrient deficiency in humans leads to a weak immune system, poor physical and intellectual development during child growth, and onset of detrimental and fatal illnesses (Bourke et al., 2016). Chemical supplementation and food fortification programs of staples such as rice with Fe and Zn have helped some governments to reduce mineral deficiencies in children, pregnant women, and low‐income community members (https://www.who.int/elena/titles/rice_fortification/en/). However, resolving hidden hunger in populations on a larger scale remains a challenge as this requires long‐term intervention strategies such as biofortification through crop breeding because of its sustainability (Bouis & Saltzman, 2017).

Rice (*Oryza sativa* L.) is a major staple crop that feeds almost 3.5 billion people worldwide. However, rice contains very low levels of mineral nutrients in the milled form (Tan et al., 2020). Upon removing the husk, the brown rice is subjected to milling to attain the desired texture and palatability (Misra et al., 2018). Unfortunately, this process leads to further significant loss of micronutrients from rice grains (Runge et al., 2019). Micronutrient concentration in milled rice is insufficient to contribute meaningfully to the daily recommended nutrient intake of micronutrients (Runge et al., 2019). Hence it is important to reveal the genetic diversity for micronutrient accumulation in brown rice to promote whole grain‐based health benefits. Assessing the genetic diversity for variation in micronutrients in brown rice can identify novel donor genotypes for essential micronutrients enriched in the bran layer of grains (Huang et al., 2015; Ravichanthiran et al., 2018).

Quantitative trait loci (QTL) mapping was used earlier to detect candidate genomic regions underlying the variation of rice micronutrients. Garcia‐Oliveira et al. (2009) used 85 introgression lines to determine QTLs for Fe, Zn, Mn, Cu, Ca, Mg, P, and K contents. Similarly, Norton et al. (2010) performed biparental mapping using Bala × Azucena, leading to the identification of 41 QTLs for 17 elements in the grains. Dixit et al. (2019) developed a BC_2_F_5_ population from RP‐Bio226 and Sampada, and identified five QTLs leading to 5–34% phenotypic variation explained (PVE) in Fe and Zn contents. Descalsota‐Empleo et al. (2019) also produced two doubled‐haploid populations from IR64 × IR69428 and BR29 × IR75862 and identified 50 QTLs (eight QTLs with >25% PVE) for 13 elements. However, these QTLs were not fine mapped to identify candidate genes. As the genetic architecture of complex traits is controlled simultaneously by many genes (Kaler & Purcell, 2019) and pinpointing important genes may be limited by mapping resolution strategies, the major effect QTLs controlling multiple mineral accumulation in rice grains remain unknown (Huang et al., 2015; Xu et al., 2015). Moreover, the QTL mapping method presents difficulties in detecting multiple genes, which simultaneously affect the complex trait of micronutrient variation in different accessions of rice (Bollinedi et al., 2020). Multigene large‐effect QTL for a trait has been described earlier (Dixit et al., 2015). Recent research deployed genome‐wide association studies (GWAS), which show polygenic inheritance for micronutrient variation as demonstrated previously (Bollinedi et al., 2020; Descalsota‐Empleo et al., 2019; Norton et al., 2010, 2014; Tan et al., 2020; Yang et al., 2018). GWAS has advantages over the biparental mapping approach, as it allows exploration of historic recombinations among the diverse natural populations to identify numerous important alleles at high resolution for different traits of interest (Islam et al., 2020).

We conducted resequencing‐based GWAS with 1.02 million high‐quality single nucleotide polymorphisms (SNPs), on a diversity panel. To complement the single marker GWAS we used gene‐level and gene set analyses (GSA; de Leeuw et al., 2015) to account for the joint effects of multiple genes. We also conducted epistasis and gene regulatory analysis using transcriptome data from the lines with contrasting haplotypes. This resulted in validating key candidate genes for the larger content of multiple minerals. The favorable alleles identified from the resequenced (RSQ) panel were used to mine for the haplotypes of existing IRRI breeding lines to identify the gaps. This study will support the filling of those gaps through biofortification and molecular breeding strategies in rice.

## RESULTS

### Phenotypic variation of an indica diversity panel and biofortified lines

**Figure 1:**
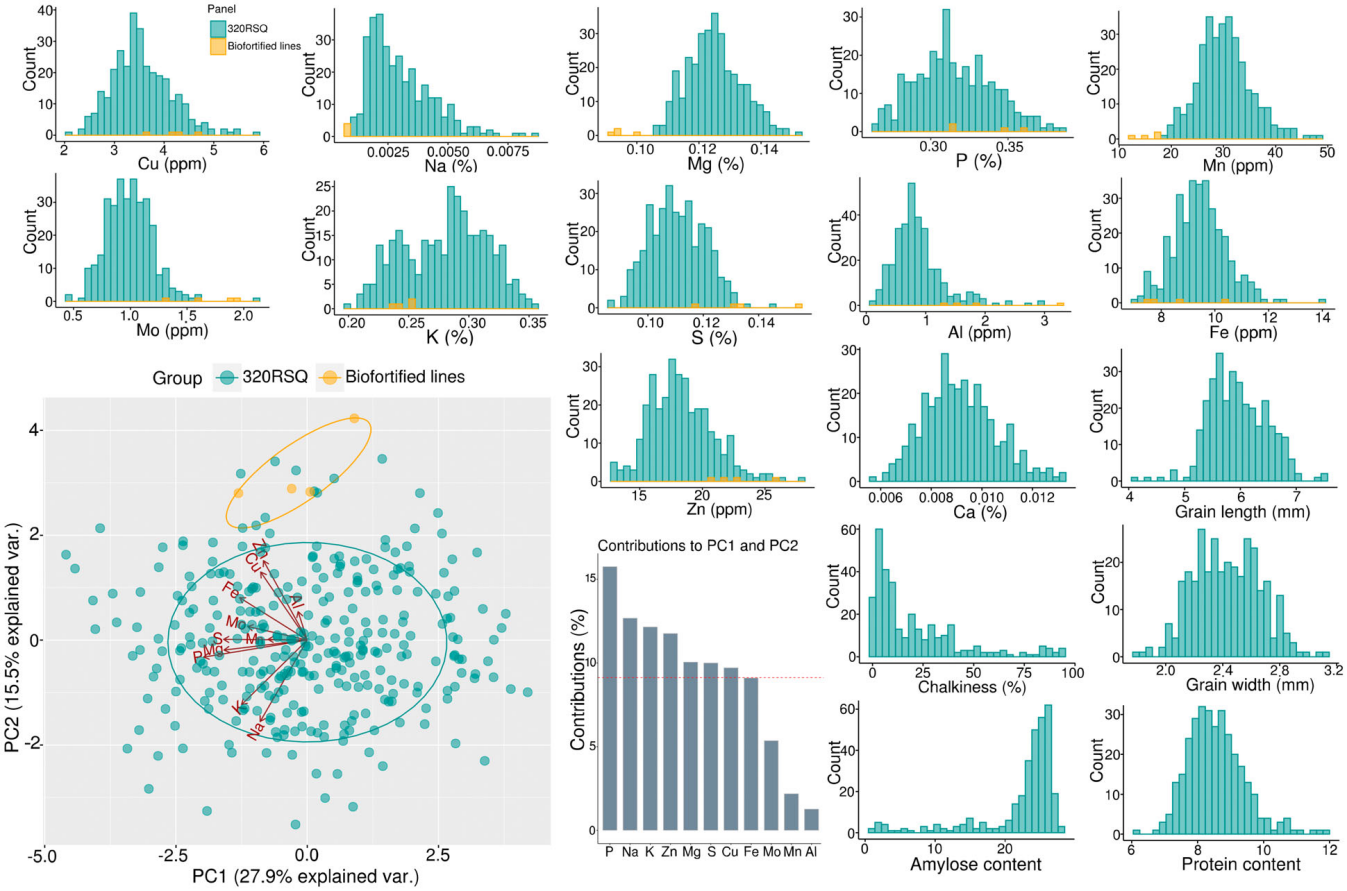
Mineral contents and grain quality traits of a diverse collection panel of *Oryza sativa* subsp. *indica*. Frequency distribution and principal components analysis for the 12 micronutrients of resequenced indica accessions (RSQ, blue color) and four IRRI breeding lines (yellow color). Also shown are the frequency distribution of five grain quality traits of the RSQ accessions.

An RSQ rice diversity panel with more than 300 *indica* accessions was used in this study to identify genomic regions linked to important micronutrients. Initially, the mineral variation of this panel was compared with four biofortified lines enriched for zinc (Zn). Except for aluminum (Al) and sodium (Na), the RSQ lines showed a wide range of phenotypic variation for most of the micronutrients in brown rice (Figure 1). The biofortified lines exhibited low concentrations of the distributions for multiple micronutrients such as Na, magnesium (Mg), and manganese (Mn). In contrast, the biofortified lines are on the right tail for Zn, molybdenum (Mo), sulfur (S), and Al. The first two principal components explained 43.4% of the total variation among the RSQ and biofortified lines, with more than 10% contributions from phosphorus (P), potassium (K), Na, and Zn. The biofortified lines had high Zn concentrations (for which they were enriched) while the RSQ lines had similar or higher levels of other micronutrients such as copper (Cu), iron (Fe), and K. The RSQ lines exhibited normal distribution for grain quality traits except chalkiness and amylose content, which were skewed to the left and right, respectively.

### Correlations among grain micronutrients and quality traits

Significant correlations (*P* < 0.05) were detected among the different micronutrients as well as grain quality traits (Figure S1). Protein content showed positive correlations with multiple micronutrients such as Mg (0.56), Mo (0.39), P (0.64), S (0.64), and Fe (0.41). Interestingly, the Zn concentration was positively correlated with Fe (0.45). Mg showed strong positive correlations with P (0.8) and moderate correlations with S (0.42), Fe (0.34), Mo (0.26), and protein (0.56). K exhibited moderate positive correlations with Na (0.46), with P (0.51), and with S (0.38). Mo correlated with P (0.4) and S (0.34). Interestingly some of the micronutrients exhibited negative correlations with grain quality traits. For instance, Mg exhibited negative correlations with chalkiness (−0.26), Mn was negatively correlated with amylose content and Ca negatively correlated with grain length.

### Genome‐wide associations targets of micronutrients

**Figure 2:**
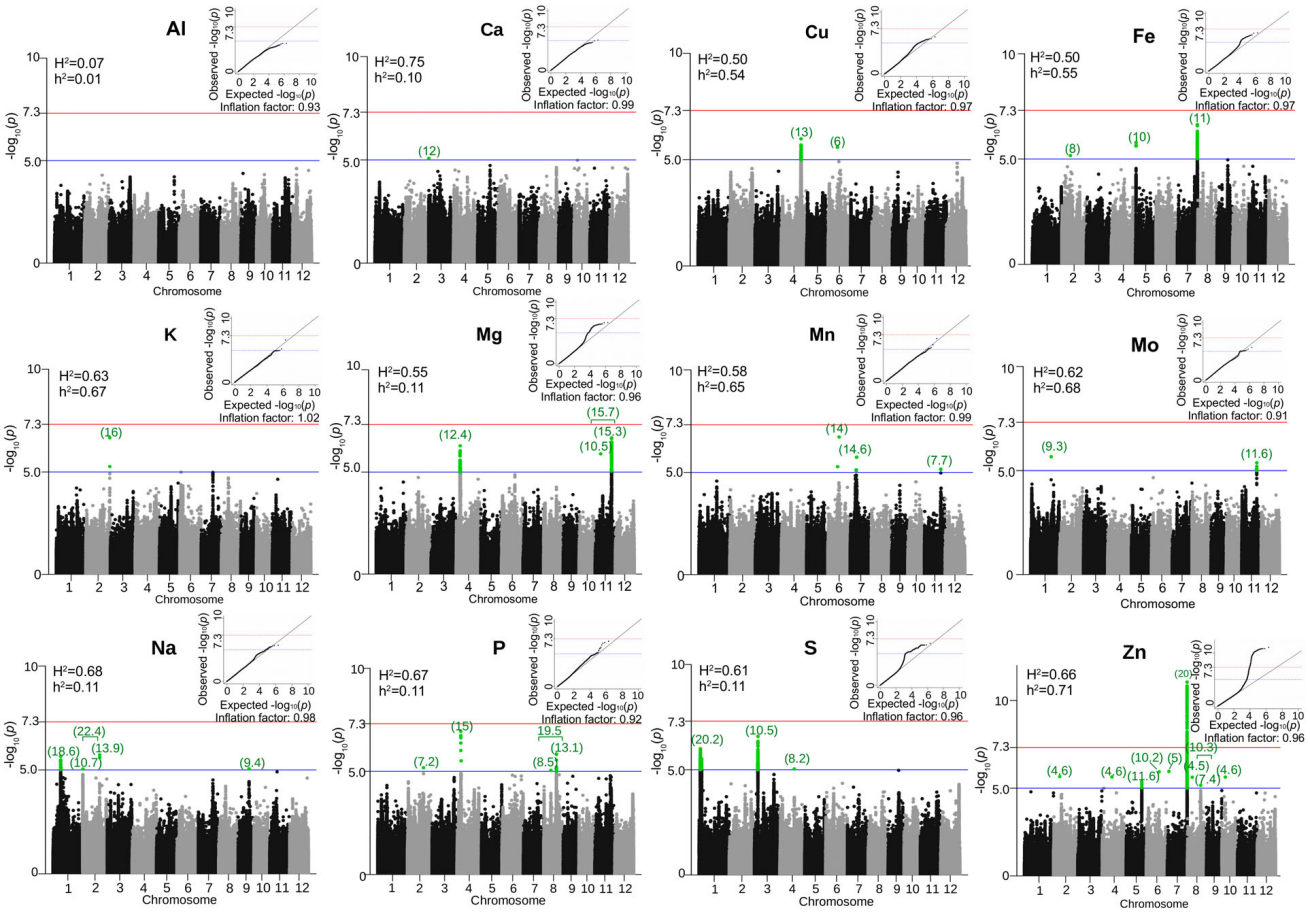
Genome‐wide associations of 12 micronutrients from a diverse panel of *Oryza sativa* subsp. *indica* RSQ accessions. These Manhattan plots reflect the results of genome‐wide association studies for each of the 12 micronutrients from RSQ panel showing significant single nucleotide polymorphism (SNPs) passing the Bonferroni cutoff (red line) or the suggestive blue line (P < 0.00001). Green numbers on top of each peak are the approximate percent*age* variation explained by the significant SNPs. Also shown in each Manhattan figure are the broad‐*sense* (*H* ^2^) and narrow‐sense (*h* ^2^) heritability values, as well as the quantile‐quantile (QQ) plots for each mineral.

The heritability values of micronutrients (except Al) were relatively good with broad‐sense heritability values ranging from 0.50 to 0.75. The narrow‐sense heritability values for Zn, Fe, Cu, K, Mn, and Mo ranged from 0.71 to 0.54 (Figure 2). Significant peaks with a total of 1120 significant (*P* < 0.00001) SNPs from GWAS analysis were detected for all the micronutrients except for Al (Figure 2; Table S1). The GWAS association peak for Zn covers 203 SNPs from chromosome 7 (approximately 20% PVE), five SNPs from chromosome 5 (approximately 12% PVE), one SNP each from chromosomes 2, 4, 6, and 10 (5–10% PVE), and two SNPs from chromosome 8 (approximately 10% PVE). Within these genetic regions several important genes were linked with Zn (*P* < 0.00001), which include *OsZIP9* (LOC_Os05g39540), *OsZIP5* (LOC_Os05g39560), and *OsNAS3* (LOC_Os07g48980), as well as *OsALDH22* (LOC_Os07g48920), *OsLonP3* (LOC_Os07g48960), *OsPOP17* (LOC_Os07g48970), *OsDLN197* (LOC_Os07g49010), *OsDLN198* (LOC_Os07g49030), *OsPP99* (LOC_Os07g49040), *OsPCS12* (LOC_Os07g49070), *OsBC1L8* (LOC_Os07g49080), *OsWD40‐154* (LOC_Os08g04290), *OsPME23* (LOC_Os07g49100), *OsSCAR4* (LOC_Os07g49140), *OsRpt2a* (LOC_Os07g49150), and *OsRH29* (LOC_Os08g32090) (Figure 2; Table S1). The important genetic region identified for Fe covers 64 SNPs from chromosome 7 with 11% PVE, one SNP from chromosome 2 (approximately 8% PVE), and two SNPs from chromosome 5 (approximately 10% PVE), including *OsNAS3* (LOC_Os07g48980) and *OsLonP3* (Figure 2; Table S1). The association peak identified for Cu includes 47 SNPs from chromosome 4 spanning more than 293 kb covering key candidate genes such as *OsHMA5* (LOC_Os04g46940) and *OsTIP5;1* (LOC_Os04g46490) with approximately 13% PVE and one exonic SNP from LOC_Os06g22240 with approximately 6% PVE (Figure 2; Table S1). The association for K linked for only two SNPs identified from different genes in chromosome 2 lying 27.5 kb apart showed 16% PVE. The association peaks identified for Mn were: six SNPs from chromosomes 6, 7, and 11 with PVE ranging from approximately 8% to approximately 15%. The association targets identified for Mo cover one downstream SNP of LOC_Os01g53830 (9% PVE) and 17 SNPs from chromosome 11 (approximately 11% PVE). Some of these candidates for Mo include *OsPR4d* (LOC_Os11g37940), *OsGAUT14* (LOC_Os11g37980), *OsCFM3* (LOC_Os11g37990), and *OsDJ‐1F* (LOC_Os11g37920) (Figure 2; Table S1).

Although the narrow sense heritability of S, Na, Mg, Ca, and P was very low, the PVE of genetic regions identified through GWAS was modest. Hence, to validate the statistical significance further, gene level analysis and GSA were performed. The GWAS association peaks for S identified 119 SNPs from chromosome 1 (approximately 7% PVE), 461 SNPs from chromosome 3 (10% PVE), and one SNP from chromosome 4 (approximately 8% PVE). The key candidate genes associated with S (*P* < 9.9309E‐06) are sulfate transporter genes such as *SULTR2;2* (LOC_Os03g09930), *SULTR2;1* (LOC_Os03g09940), *SULTR1;1* (LOC_Os03g09970), and *SULTR1;2* (LOC_Os03g09980), and *OsALD1* (LOC_Os03g09910). Other SNPs associated with S include one intronic SNP from *OsALMT1* (LOC_Os04g34010) and more SNPs were located mostly on chromosome 1 (1.9 Mb) overlapping genes *FIB* (LOC_Os01g07500), *OsSTE1* (LOC_Os01g04260), and *OsCRS2* (LOC_Os01g04130) (Figure 2; Table S1). The genetic regions identified for Na cover 14 SNPs from chromosome 1 (approximately 19% PVE), chromosome 2 (approximately 22% PVE), and chromosome 9 (approximately 9% PVE) covering key candidate genes including *NAC51* (LOC_Os02g41450) and *OsAlaAT5* (LOC_Os09g26380) (Figure 2; Table S1). The GWAS peaks for Mg identified 87 SNPs from chromosome 11 spanning almost 589 kb with approximately 16% PVE, and 59 SNPs spanning about 654.8 kb in chromosome 4 with 12% PVE. Most SNPs associated with Mg have a beta effect (β) >0.3075, while one exonic SNP from LOC_Os11g39780 showed β = 0.5277. The significant genetic regions for Ca identified only one SNP upstream of *OsEXPB8* (LOC_Os03g01260) with a PVE of approximately 11%, and for P, four SNPs from chromosome 4 (approximately 15% PVE), one SNP from chromosome 2 (approximately 7% PVE), and 15 SNPs from chromosome 8 (approximately 19% PVE) (Figure 2; Table S1).

### Key candidate genes identified for rice micronutrients through gene‐level analysis and GSA

Gene‐level analysis and GSA confirmed key candidate genes found through GWAS and revealed additional candidate genes not detected through GWAS (Table S2). For S, all top candidate genes identified from GSA were also found in GWAS analysis, which includes sulfate transporters *SULTR2;2* (LOC_Os03g09930), *SULTR2;1* (LOC_Os03g09940), *SULTR1;1* (LOC_Os03g09970), and *SULTR1;2* (LOC_Os03g09980), and *OsALMT1* (LOC_Os04g34010). Similarly for Zn, five of six genes (*OsLonP3*/LOC_Os07g48960, *OsPOP17* [LOC_Os07g48970], and *OsALDH22*/LOC_Os07g48920, and two retrotransposons) validated from the top gene set were also detected through GWAS. For Na, two candidate genes (*OsAlaAT5/*LOC_Os09g26380 and LOC_Os02g01500) validated through the top gene set were also found in GWAS. Other candidate genes from GSA for Na encode OsHKT8/OsHKT1 (LOC_Os01g20160) and different kinds of enzymes. For Cu, four candidate genes including *HMA5/OsHMA8/* (LOC_Os04g46940), *OsTIP5;1* (LOC_Os04g46490), and *OsEXPB5* (LOC_Os04g46650) identified through GWAS were also part of the top gene set consisting of seven other candidate genes encoding Zn knuckle (LOC_Os04g46920), AP2 (LOC_Os04g46250), WD (LOC_Os04g46892), glutathione peroxidase (LOC_Os04g46960), and Zn finger (LOC_Os04g46600). For Mg, six candidate genes identified from GWAS were also found in the top gene set based on GSA, with most candidates encoding numerous enzymes such as cysteine proteases (LOC_Os01g13920, LOC_Os01g67350), phospholipase (LOC_Os11g40009), and ent‐kaurene synthase (LOC_Os04g10060), along with DEFL80 (LOC_Os11g39910) and patatin (LOC_Os11g39990). In the case of Mn, only one candidate gene from GWAS was found in the top gene set, which includes eight other candidate genes having a β = 1.8 at *P* < 0.001. Similarly, only one SNP was linked with Ca from GWAS but eight candidate genes showed joint association for Ca based on GSA, which includes *OsCIPK29* (LOC_Os07g48090: encodes a calcium/calmodulin‐dependent protein kinase). While only one candidate from GWAS overlapped with GSA results for P, three candidate genes from top gene set for P were linked with Mg and one candidate gene was linked with Mo based on GWAS. The top set of candidate genes for P mostly encodes for enzymes such as amidase (LOC_Os04g10410), hydroxyacid oxidase 1 homologs (LOC_Os04g53214, LOC_Os04g53210), glucose‐6‐phosphate isomerase (LOC_Os03g56460), aminomethyltransferase (LOC_Os04g53230), and 1‐aminocyclopropane‐1‐carboxylate oxidase homolog 1 (LOC_Os08g30240). Al was not linked with any genes based on GWAS but was linked with eight candidate genes from GSA, including *OsHK5* (LOC_Os10g21810) and *OsABCI14* (LOC_Os02g56550).

### Association networks highlighting key candidate genes influencing multiple micronutrients in rice grains

**Figure 3:**
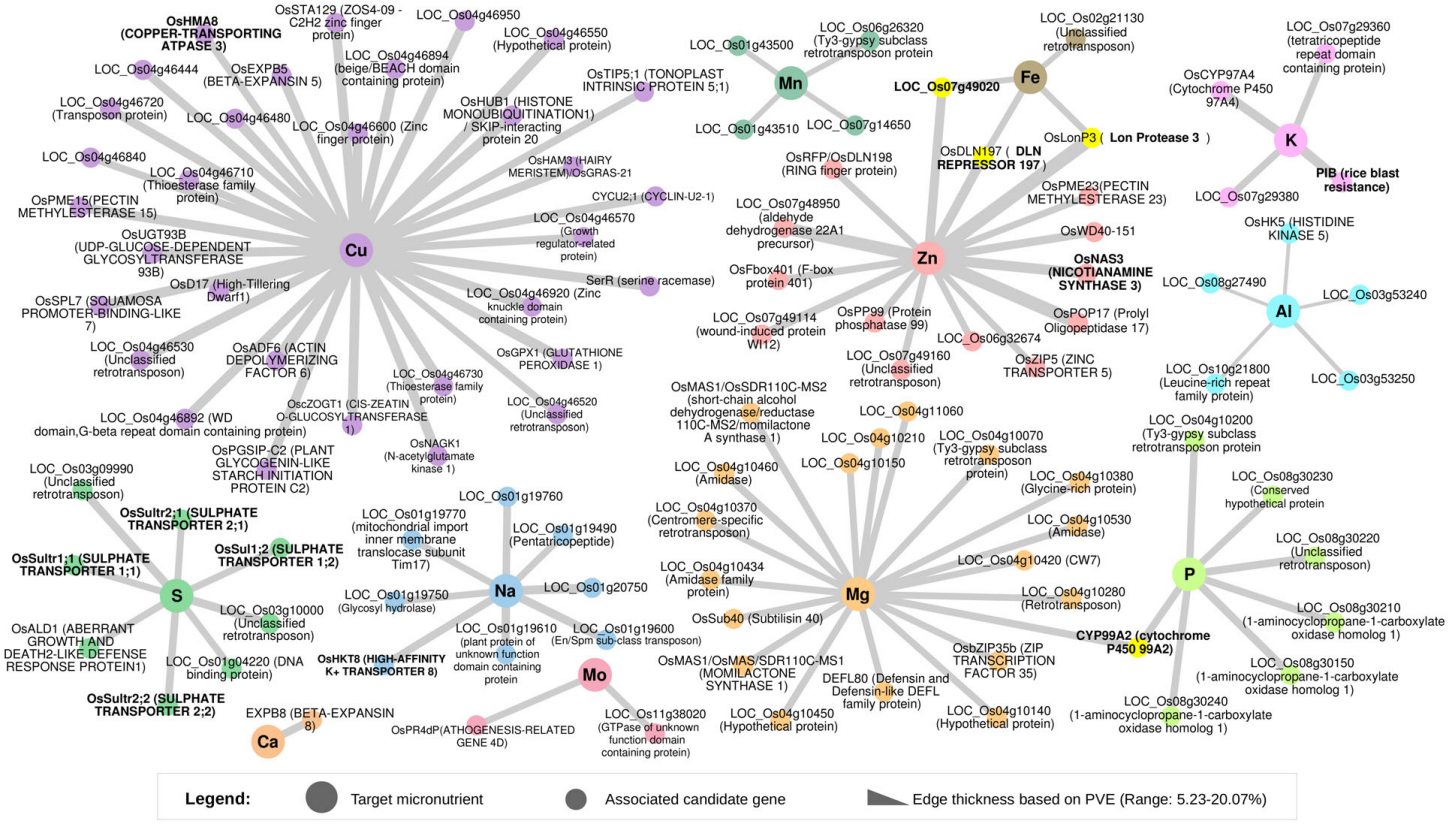
Association network summary of candidate genes linked with the 12 micronutrients of a diverse panel of *Oryza sativa* subsp. *indica* RSQ accessions. *C*entral nodes represent the micronutrients of interest and the smaller nodes are the associated candidate genes, while the edge thickness (gray line) represents the percentage variation explained value (PVE) of the candidate gene (range: 5.2–20.1%). These candidate genes were determined through targeted *association analysis after genome‐wide association studies* and gene set analyses, with their top non‐redundant single nucleotide polymorphisms filtered based on Bonferroni cutoff and beta effect values (β > 2.0 or β < −2.0). Further filtering was performed based on the PVE range of candidate genes per mineral with the following thresholds: PVE >8% was used for Fe and Mn, >5% for Al, and >10% for Cu, S, Na, Mg, P, Zn, Ca, and K.

**Figure 4:**
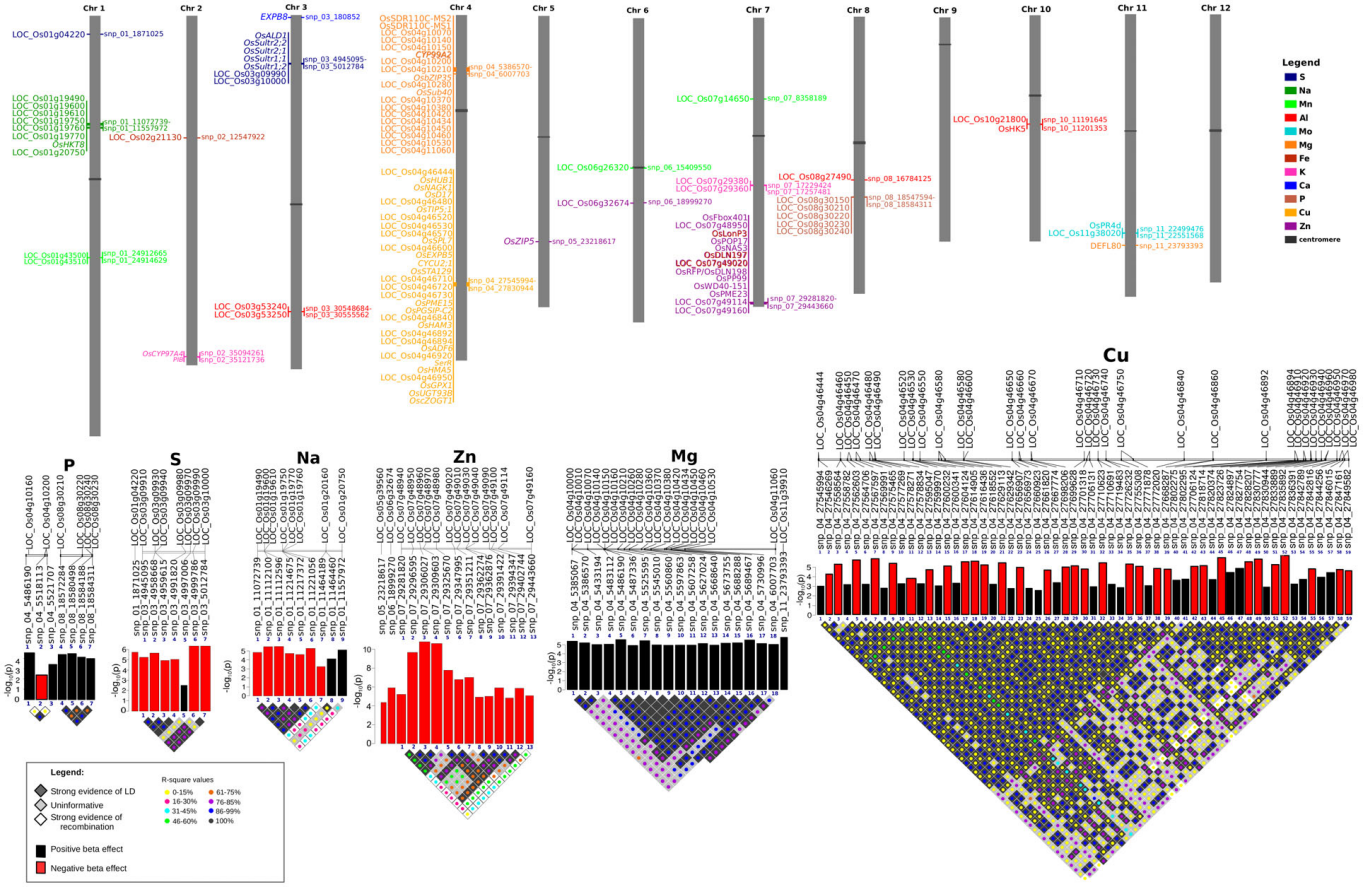
Chromosomal positions of the candidate genes linked with the 12 micronutrients of a diverse panel of *Oryza sativa* subsp. *indica* RSQ accessions. Top candidate genes per mineral are mapped to corresponding chromosome positions. In addition, the linkage maps of tag single nucleotide polymorphisms (SNPs) from the candidate genes per mineral based on 95% confidence intervals on D′ (background color of diamond pattern represents *LD*, while colored circles inside each diamond pattern represent *r* ^2^ values for each SNP pair). The corresponding –log_10_ P values and beta effect of the SNPs are also shown (bar width represents absolute beta effects: thicker bar = higher absolute beta effect). LD, linkage disequilibrium.

All the candidate genes from GWAS, GSA, and known genes reported from model species in the literature were then used for targeted associations to identify causal SNPs per gene. From almost 300 candidate genes, there were more than 3300 SNPs associated with 12 micronutrients from RSQ lines (Table S3). By excluding redundant SNPs and filtering the candidate genes based on a stringent PVE threshold explained by the top SNPs, the number of candidate genes narrowed down to 109 with PVE ranging from 5% to 20% (Figure 3; Table S4). Through association networks, interesting key candidate genes *OsLonP3/*LOC_Os07g48960, *OsDLN197*/LOC_Os07g49010, and LOC_Os07g49020 were identified that influence multiple traits such as Fe and Zn on chromosome 7 (Figure 3). The candidate gene *CYP99A2* (LOC_Os04g10160) associated with Mg and P (Figure 3). Multiple candidate genes explaining higher PVE were located at a “hot‐spot” QTL region on chromosome 4 for Cu, Mg, Na, and P (Figure 4). For S, many sulfur transporters, which are tandemly duplicated were identified within the QTL region at the upper arm of chromosome 3 (Figure 4). Zooming into the linkage disequilibrium (LD) plots, low *r* ^2^ values were usually found between different pairs of SNPs within one LD block, suggesting that multiple target genes identified within the hot‐spot QTL region might be necessary to elevate the micronutrient concentration (Figure 4).

### High value genomic variants explaining higher PVE with genome‐wide combined multi‐haplotypes defined across chromosomes

Interestingly, the total PVE based on top non‐redundant SNPs identified from the multiple candidate genes across different chromosomes exhibited higher values compared with individual PVEs per candidate gene (Table 1). For instance, in the case of Zn, individual candidate genes showed 10–20% PVE, but when taken together from genome‐wide combined multi‐haplotypes, the total PVE was 39.32%. For Fe, the individual candidate genes explained 8–9% phenotypic variation while the genome‐wide combined multi‐haplotypes derived from multiple candidate genes resulted in 18.78% PVE. Notably, three candidate genes for Fe also overlapped with Zn candidates on chromosome 7 and near the QTL *qAOC_IPR7.1* previously detected with polished rice (Suman et al., 2021). The candidate genes for Mn showed about 8% PVE but when taken together explained almost 20% phenotypic variation. Similar results were observed in other elements such as Cu, K, P, S, Na, and Mg where the total PVE was approximately double the PVE of individual candidate genes. The top non‐redundant SNPs across the candidate genes resulted from combined multi‐haplotypes per micronutrient were then used to form the superior or inferior functional haplotypes explaining higher or lower levels of micronutrients, respectively.

**Table 1:** List of candidate genes with highest percentage variation explained (PVE) values for each of the 12 micronutrients of the diverse panel of *Oryza sativa* subsp*. indica* RSQ lines identified through genome‐wide association studies, gene set analysis, and gene‐targeted association

Trait	Target genomic region	Gene	Protein	PVE (%)	Top snps	Total PVE (%)	Superior haplotype	Inferior haplotype	References related to possible gene function or overlapping QTL previously reported
Al	*qAl3.1*	LOC_Os03g53240	LOC_Os03g53240 (expressed protein)	5.28	snp_03_30548939, snp_03_30555562, snp_08_16784125, snp_10_11191645, snp_10_11201353	14.37	TGGCA	CCTTG	
		LOC_Os03g53250	LOC_Os03g53250 (expressed protein)	6.84					
	*qAl8.1*	LOC_Os08g27490	LOC_Os08g27490 (expressed protein)	5.23					
	*qA10.1*	LOC_Os10g21800	LOC_Os10g21800 (Leucine‐rich repeat family protein)	5.78					
		LOC_Os10g21810	OsHK5 (HISTIDINE KINASE 5)	5.55					
Cu	*qCu4.1*	LOC_Os04g46444	LOC_Os04g46444 (expressed protein)	12.66	snp_04_27545994, snp_04_27546269, **snp_04_27558782**, **snp_04_27569991**, snp_04_27578271, snp_04_27578834, snp_04_27599970, snp_04_27600232, **snp_04_27600341**, snp_04_27604124, snp_04_27614905, snp_04_27616436, snp_04_27618552, **snp_04_27629342,** snp_04_27656973, **snp_04_27660923, snp_04_27667774**, snp_04_27668287, **snp_04_27701318**, snp_04_27706124, snp_04_27706131, **snp_04_27710623,** snp_04_27710691, **snp_04_27726232,** snp_04_27755308, snp_04_27771878, snp_04_27772020, snp_04_27802275, snp_04_27802295, **snp_04_27820374,** snp_04_27823726, **snp_04_27827754,** snp_04_27828207, snp_04_27830777, **snp_04_27830944,** snp_04_27833889, snp_04_27835892, snp_04_27836391, **snp_04_27842789**, snp_04_27842816, **snp_04_27844256, snp_04_27846015,** snp_04_27847161, snp_04_27849582	25.44	**A**G**AG**GACA**C**GCCG**C**G**TA**A**T**GT**T**C**G**GGCCCAA**G**C**GG**CCG**A**G**CC**CA	**G**G**GA**GCTA**T**GCCA**T**A**CG**A**C**GA**G**C**A**GGTTCGA**A**C**GT**CCA**G**G**AT**CA	
	*qCu4.2*	LOC_Os04g46450	OsHUB1 (HISTONE MONOUBIQUITINATION1) / SKIP‐interacting protein 20	15.22					
		LOC_Os04g46460	OsNAGK1 (*N*‐acetylglutamate kinase 1)	14.41					
		LOC_Os04g46470	OsD17 (High‐Tillering Dwarf1)	15.20					
		LOC_Os04g46480	LOC_Os04g46480 (expressed protein)	14.75					
		LOC_Os04g46490	OsTIP5;1 (TONOPLAST INTRINSIC PROTEIN 5;1)	11.41					
		LOC_Os04g46520	LOC_Os04g46520 (Unclassified retrotransposon)	10.51					
		LOC_Os04g46530	LOC_Os04g46530 (Unclassified retrotransposon)	14.34					
		LOC_Os04g46550	LOC_Os04g46550 (Hypothetical protein)	14.81					
		LOC_Os04g46570	LOC_Os04g46570 (Growth regulator‐related protein)	11.66					
		LOC_Os04g46580	OsSPL7 (SQUAMOSA PROMOTER‐BINDING‐LIKE 7)	15.09					
		LOC_Os04g46600	LOC_Os04g46600 (zinc finger protein)	14.17					
		LOC_Os04g46650	OsEXPB5 (BETA‐EXPANSIN 5)	13.50					
		LOC_Os04g46660	CYCU2;1 (CYCLIN‐U2‐1)	12.61					
		LOC_Os04g46670	OsSTA129 (ZOS4‐09 ‐ C2H2 zinc finger protein)	14.21					
		LOC_Os04g46710	LOC_Os04g46710 (thioesterase family protein)	11.53					
		LOC_Os04g46720	LOC_Os04g46720 (transposon protein)	13.09					
		LOC_Os04g46730	LOC_Os04g46730 (thioesterase family protein)	14.16					
		LOC_Os04g46740	OsPME15 (PECTIN METHYLESTERASE 15)	14.08					Krzesłowska (2011)
		LOC_Os04g46750	OsPGSIP‐C2 (PLANT GLYCOGENIN‐LIKE STARCH INITIATION PROTEIN C2)	14.48					
		LOC_Os04g46840	LOC_Os04g46840 (expressed protein)	10.95					
	*qCu4.3*	LOC_Os04g46860	OsHAM3 (HAIRY MERISTEM)/OsGRAS‐21	13.23					
	*qCu4.4*	LOC_Os04g46892	LOC_Os04g46892 (WD domain, G‐beta repeat domain containing protein)	13.87					
	*qCu4.5*	LOC_Os04g46894	LOC_Os04g46894 (beige/BEACH domain containing protein)	14.05					
		LOC_Os04g46910	OsADF6 (actin DEPOLYMERIZING FACTOR 6)	16.57					Inada (2017)
		LOC_Os04g46920	LOC_Os04g46920 (zinc knuckle domain containing protein)	16.40					
		LOC_Os04g46930	SerR (serine racemase)	14.59					
		LOC_Os04g46940	**OsHMA5 (Copper‐transporting ATPase HMA5/COPPER‐TRANSPORTING ATPASE 3)**	11.46					Deng et al. (2013)
		LOC_Os04g46950	LOC_Os04g46950 (expressed protein)	13.67					
		LOC_Os04g46960	OsGPX1 (GLUTATHIONE PEROXIDASE 1)	15.40					
		LOC_Os04g46970	OsUGT93B (UDP‐GLUCOSE‐DEPENDENT GLYCOSYLTRANSFERASE 93B)	13.57					
	*qCu4.6*	LOC_Os04g46980	OscZOGT1 (CIS‐ZEATIN O‐GLUCOSYLTRANSFERASE 1)	14.32					
Fe	*qFe2.1*	LOC_Os02g21130	LOC_Os02g21130 (unclassified retrotransposon)	8.21	snp_02_12547922, snp_07_29298014, snp_07_29306027, snp_07_29347995, snp_07_29351211	18.78	TCCTT	TTTCC	
	*qFe7.1*	LOC_Os07g48960	OsLonP3 (Lon Protease 3)	9.97					Near the QTL *qAOC_IPR7.1* for Fe in polished rice detected by Suman et al. (2021)
	*qFe7.2*	LOC_Os07g49010	OsDLN197 (DLN REPRESSOR 197)	9.92					
		LOC_Os07g49020	LOC_Os07g49020 (expressed protein)	8.67					
Mn	*qMn1.1*	LOC_Os01g43500	LOC_Os01g43500 (expressed protein)	8.28	snp_01_24912665, snp_06_15409550, snp_07_8358189	19.99	CCG	CTA	
		LOC_Os01g43510	LOC_Os01g43510 (expressed protein)	8.12					
	*qMn6.1*	LOC_Os06g26320	LOC_Os06g26320 (Ty3‐gypsy subclass retrotransposon protein	8.33					
	*qMn7.1*	LOC_Os07g14650	LOC_Os07g14650 (expressed protein)	8.12					Near a QTL for Mn (lead SNP at 9.1 Mb) in Chr 7 detected in two locations at Youxian by Yang et al. (2018)
Mo	*qMo11.1*	LOC_Os11g37940	OsPR4dP(ATHOGENESIS‐RELATED GENE 4D)	10.12	snp_11_22499476, snp_11_22500044, snp_11_22551568	12.43	CCC or CTC	ACT	
		LOC_Os11g38020	LOC_Os11g38020 (GTPase of unknown function domain containing protein)	10.41					
Zn	*qZn5.1*	LOC_Os05g39560	OsZIP5 (ZINC TRANSPORTER 5)	10.04	snp_05_23218617, snp_06_18999270, snp_07_29281820, snp_07_29296595, snp_07_29306027, snp_07_29309603, snp_07_29325670, snp_07_29347995, snp_07_29351211, snp_07_29362876, snp_07_29391422, snp_07_29394347, snp_07_29402744, snp_07_29443660	39.32	ACGGTCTCCTGTTA	CAGGTCTCCTGTTA	*OsZIP5* (Lee et al., 2010)
	*qZn6.1*	LOC_Os06g32674	LOC_Os06g32674 (hypothetical protein)	10.19					
	*qZn7.1*	LOC_Os07g48940	OsFbox401 (F‐box protein 401)	10.73					*OsNAS3* (Aung et al., 2019); *qZn7.2* in four environments
		LOC_Os07g48950	LOC_Os07g48950 (aldehyde dehydrogenase 22A1 precursor)	18.44					
		LOC_Os07g48960	OsLonP3 (Lon protease 3)	20.07					
		LOC_Os07g48970	OsPOP17 (prolyl oligopeptidase 17)	19.67					
		LOC_Os07g48980	OsNAS3 (NICOTIANAMINE SYNTHASE 3)	15.35					
		LOC_Os07g49010	OsDLN197 (DLN REPRESSOR 197)	14.07					
		LOC_Os07g49020	LOC_Os07g49020 (expressed protein)	13.58					
	*qZn7.2*	LOC_Os07g49030	OsRFP/OsDLN198 (RING finger protein, DLN repressor 198)	10.59					
		LOC_Os07g49040	OsPP99 (protein phosphatase 99)	10.69					
		LOC_Os07g49090	OsWD40‐151 (pectinesterase)	12.32					
		LOC_Os07g49100	OsPME23 (PECTIN METHYLESTERASE 23)	10.30					
		LOC_Os07g49114	LOC_Os07g49114 (wound‐induced protein WI12)	12.22					
	*qZn7.3*	LOC_Os07g49160	LOC_Os07g49160 (unclassified retrotransposon)	10.69					*qZn7.3* in one environment
Ca	*qCa3.1*	LOC_Os03g01260	EXPB8 (BETA‐EXPANSIN 8)	11.55	snp_03_180852	11.55	C	T	Marowa et al. (2016)
K	*qK2.1*	LOC_Os02g57290	OsCYP97A4 (cytochrome P450 97A4)	13.43	snp_02_35094261, snp_02_35121736, snp_07_17257481	25.51	AAC	GGG	
		LOC_Os02g57310	PIB (rice blast resistance)	14.45					Roychowdhury et al. (2012)
	*qK7.1*	LOC_Os07g29360	LOC_Os07g29360 (tetratricopeptide repeat domain containing protein)	10.47					
		LOC_Os07g29380	LOC_Os07g29380 (expressed protein)	10.47					
P	*qP4.1*	LOC_Os04g10160	CYP99A2 (cytochrome P450 99A2)	10.90	snp_04_5486190, snp_04_5518113, snp_04_5521707, snp_08_18572284, snp_08_18580498, snp_08_18584188, snp_08_18584311	24.05	GAAACCG	GAAGTTT	Shimura et al. (2007)
	*qP4.2*	LOC_Os04g10200	LOC_Os04g10200 (Ty3‐gypsy subclass retrotransposon protein	10.97					
	*qP8.1*	LOC_Os08g30150	LOC_Os08g30150 (1‐aminocyclopropane‐1‐carboxylate oxidase homolog 1)	11.75					Houben and Van de Poel (2019)
		LOC_Os08g30210	LOC_Os08g30210 (1‐aminocyclopropane‐1‐carboxylate oxidase homolog 1)	10.89					
		LOC_Os08g30220	LOC_Os08g30220 (unclassified retrotransposon)	11.47					
		LOC_Os08g30230	LOC_Os08g30230 (conserved hypothetical protein	10.38					
		LOC_Os08g30240	LOC_Os08g30240 (1‐aminocyclopropane‐1‐carboxylate oxidase homolog 1)	10.98					
S	*qS1.1*	LOC_Os01g04220	LOC_Os01g04220 (DNA binding protein)	10.43	snp_01_1871025, snp_03_4958668, snp_03_4991820, snp_03_4997006, snp_03_5012784	24.72	CCTGA	TTCTG	
	*qS3.1*	LOC_Os03g09910	OsALD1 (ABERRANT GROWTH AND DEATH2‐LIKE DEFENSE RESPONSE PROTEIN1)	10.55					
		LOC_Os03g09930	OsSultr2;2 (SULFATE TRANSPORTER 2;2)	11.35					Takahashi (2010)
		LOC_Os03g09940	OsSultr2;1 (SULFATE TRANSPORTER 2;1)	10.13					
	*qS3.2*	LOC_Os03g09970	OsSultr1;1 (SULFATE TRANSPORTER 1;1)	10.25					Kumar et al. (2019)
		LOC_Os03g09980	OsSul1;2 (SULFATE TRANSPORTER 1;2)	10.29					
	*qS3.3*	LOC_Os03g09990	LOC_Os03g09990 (unclassified retrotransposon)	12.45					
		LOC_Os03g10000	LOC_Os03g10000 (unclassified retrotransposon)	12.45					
Na	*qNa1.1*	LOC_Os01g19490	LOC_Os01g19490 (pentatricopeptide)	10.28	snp_01_11072739, snp_01_11112100, snp_01_11217372, snp_01_11221056, snp_01_11464189, snp_01_11464460, snp_01_11557972	17.93	TTCTGGG	CGTAAAA	*qNa1.2* in two environments
		LOC_Os01g19600	LOC_Os01g19600 (CACTA, En/Spm sub‐class transposon protein	12.38					
		LOC_Os01g19610	LOC_Os01g19610 (plant protein of unknown function domain containing protein	12.51					
		LOC_Os01g19750	LOC_Os01g19750 (glycosyl hydrolase)	10.15					
		LOC_Os01g19760	LOC_Os01g19760 (expressed protein)	11.29					
		LOC_Os01g19770	LOC_Os01g19770 (mitochondrial import inner membrane translocase subunit Tim17)	10.12					
	*qNa1.2*	LOC_Os01g20160	OsHKT8 (HIGH‐AFFINITY K+ TRANSPORTER 8)	11.61					*qNa1.2* in two environments (Kobayashi et al., 2017); near a QTL for Na (lead SNP at 11.48 Mb) detected in Wuhan by Yang et al. (2018); *qNA1* by Descalsota‐Empleo et al. (2019)
	*qNa1.3*	LOC_Os01g20750	LOC_Os01g20750	13.12					
Mg	*qMg4.1*	LOC_Os04g10000	OsMAS1/OsSDR110C‐MS2 (short‐chain alcohol dehydrogenase/reductase 110C‐MS2/momilactone A synthase 1)	10.84	snp_04_5385067, snp_04_5433194, snp_04_5487336, snp_04_5597863, snp_04_5668640, snp_04_5673755, snp_04_5689467, snp_04_5730996, snp_04_6007703, snp_11_23793393	22.13	CAACGTGTCT	GGGTACACTT	
		LOC_Os04g10010	OsMAS1/OsMAS/SDR110C‐MS1 (MOMILACTONE SYNTHASE 1)	10.70					
		LOC_Os04g10070	LOC_Os04g10070 (Ty3‐gypsy subclass retrotransposon protein)	10.32					
	*qMg4.2*	LOC_Os04g10140	LOC_Os04g10140 (hypothetical protein)	10.13					
		LOC_Os04g10150	LOC_Os04g10150 (expressed protein)	10.21					
		LOC_Os04g10160	CYP99A2 (cytochrome P450 99A2)	11.05					Shimura et al. (2007)
		LOC_Os04g10210	LOC_Os04g10210 (expressed protein)	10.90					
		LOC_Os04g10260	OsbZIP35 (bZIP TRANSCRIPTION FACTOR 35)	10.16					
		LOC_Os04g10280	LOC_Os04g10280 (retrotransposon)	10.06					
		LOC_Os04g10360	OsSub40 (Subtilisin 40)	10.12					
		LOC_Os04g10370	LOC_Os04g10370 (centromere‐specific retrotransposon)	10.04					
		LOC_Os04g10380	LOC_Os04g10380 (glycine‐rich protein)	10.38					
		LOC_Os04g10420	LOC_Os04g10420 (CW7)	10.04					
		LOC_Os04g10434	LOC_Os04g10434 (amidase family protein)	10.33					
		LOC_Os04g10450	LOC_Os04g10450 (hypothetical protein)	10.64					
		LOC_Os04g10460	LOC_Os04g10460 (amidase)	11.34					
		LOC_Os04g10530	LOC_Os04g10530 (amidase)	10.53					
	*qMg4.3*	LOC_Os04g11060	LOC_Os04g11060 (expressed protein)	10.40					
	*qMg11.1*	LOC_Os11g39910	DEFL80 (defensin and defensin‐like DEFL family protein)	10.30					

Non‐redundant SNPs filtered and used to form the functional haplotype for copper (Cu) (in bold).

PVE, percentage variation explained; QTL, quantitative trait loci; SNP, single nucleotide polymorphism.

### Genome‐wide SNP variants exhibiting epistatic interactions with multiple micronutrients

**Figure 5:**
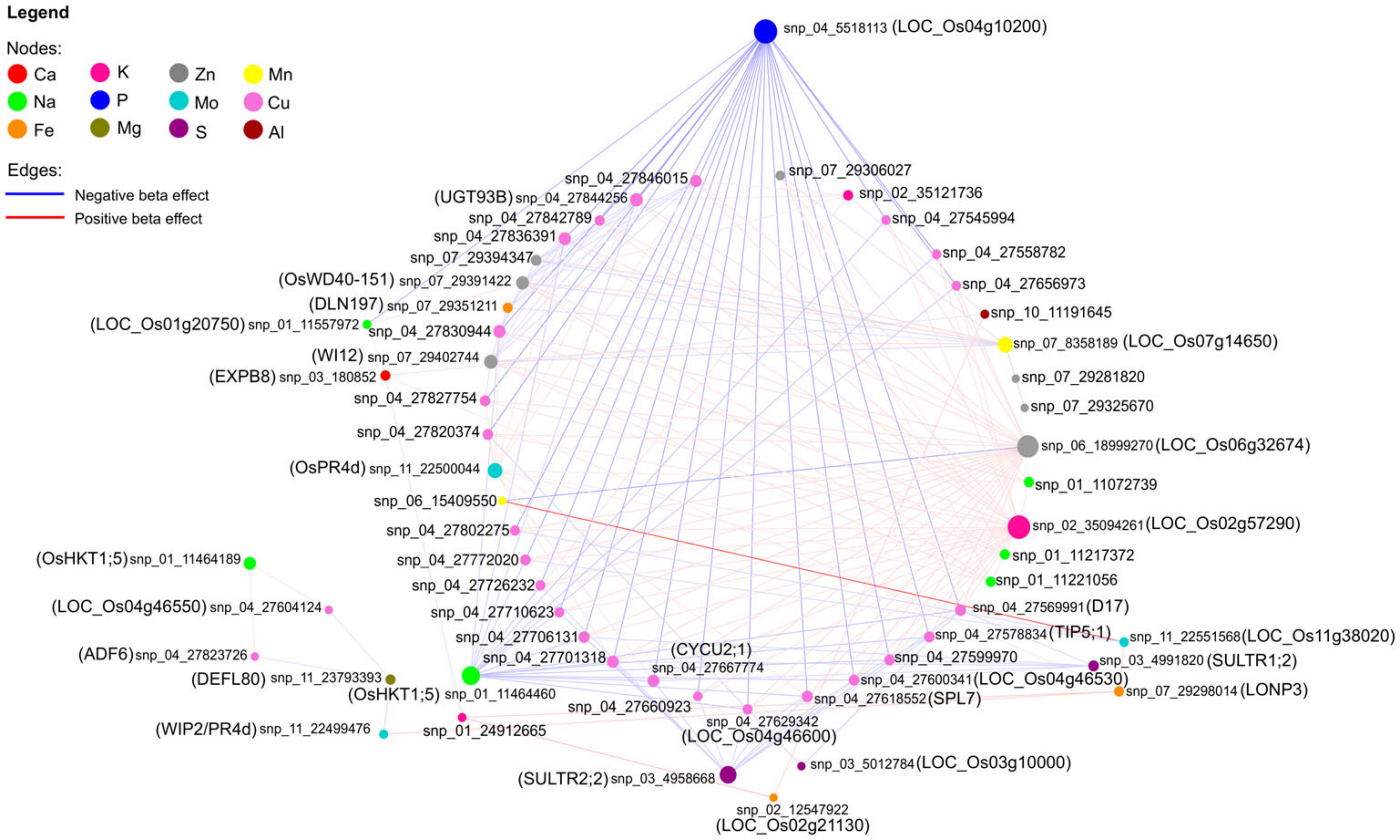
Epistatic interactions of the top non‐redundant SNPs from the candidate genes for various micronutrients of a diverse panel of *Oryza sativa* subsp. *indica* RSQ accessions. These SNPs from the candidate genes for different micronutrients specified in parentheses showed significant pairwise interactions *P* < 0.001 with at least two other SNPs and were also epistatically linked with other micronutrients color based on legend. Edge colors: red for positive beta effect, and blue for negative beta effect; node size signifies number of interactors. SNP, single nucleotide polymorphism.

Except for one SNP, all significant top non‐redundant SNPs (103) linked with the 12 micronutrients showed epistatic interactions with at least two other significant SNPs at *P* < 0.001 (Figure 5). In total, 213 epistatic interactions were observed among the significant SNPs linked to the micronutrients (Table S5). From all the epistatic interactions of the top SNPs, there were six major SNPs with at least 14 epistatic interactions with other SNPs. The higher epistatic interaction includes a synonymous variant (snp_04_5518113, big blue node) in LOC_Os04g10200 encoding retrotransposon‐encoding gene linked to P, which showed negative epistatic interactions to multiple SNPs associated mostly with Cu, and to an SNP (snp_01_11557972) linked with Na. Similarly, an upstream SNP (snp_01_11464460: big bright green node) of a Na/K transporter (OsHKT1;5) linked with Na showed negative epistatic interaction with multiple SNPs linked to Cu. Two upstream variants, snp_03_4958668 (big purple node) and snp_03_4991820 (small purple node) of the sulfate transporters SULTR2;2 and SULTR1;2 showed negative epistatic interactions with SNPs linked to Cu. On the other hand, a downstream SNP (snp_07_8358189, big yellow node) of LOC_Os07g14650 linked with Mn exhibited epistatic interactions with an SNP (snp_07_29351211) linked with Fe and with multiple SNPs linked with Zn. The SNP, snp_06_18999270 (big gray node), which is located upstream of LOC_Os06g32674 (hypothetical protein) linked to Zn showed positive epistatic interactions with multiple SNPs linked to Cu, but negative epistatic interaction (−0.84) with a splice acceptor variant (snp_06_15409550) of LOC_Os06g26320 (Ty3‐gypsy subclass retrotransposon), which was linked with Mn. In contrast, this splice acceptor variant showed a positive epistatic interaction with an upstream SNP (snp_11_22551568) of LOC_Os11g38020 linked with Mo with a high β (1.71). In addition, a synonymous variant (snp_02_35094261) of LOC_Os02g57290 encoding a cytochrome P450 protein linked with K was observed to have positive epistatic interactions with multiple SNPs linked to Cu. Moreover, a significant SNP (snp_07_29298014) of *LonP3* conferring a change in amino acid linked to Fe showed positive epistatic interactions with a splice region variant (snp_11_22499476) of *OsPR4d* and an upstream SNP (snp_11_22551568) of LOC_Os11g38020 linked to Mo, downstream SNP (snp_01_24912665) of LOC_Os01g43500 linked to K, and an upstream SNP (snp_03_180852) of *EXPB8* linked with Ca. Another SNP (snp_02_12547922) linked to Fe also showed positive epistatic interaction with a downstream SNP (snp_10_11191645) of LOC_Os10g21800 linked to Al but this downstream SNP was negatively epistatic to an SNP (snp_02_35121736) upstream of LOC_Os02g57310 linked to K. Interestingly, this upstream SNP showed positive epistatic interactions to various SNPs linked with Cu, Zn, and Na.

### Coexpression transcriptomic network analysis of differentially regulated central hub genes strongly associated with micronutrients

**Figure 6:**
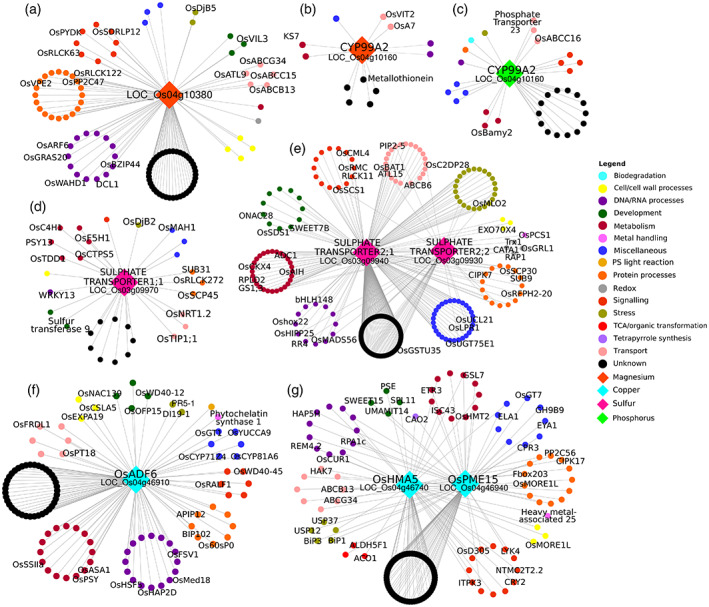
Differentially expressed candidate genes for various micronutrients of contrasting *Oryza sativa* subsp. *indica* RSQ lines along with their coexpressed genes. Candidate genes for Mg (a, b), P (c), S (d, e), and Cu (f, g) were found to be differentially expressed among RSQ lines with contrasting levels of corresponding micronutrients based on transcriptome data of developing seeds (16 days after fertilization). First‐neighbor coexpressed genes for each candidate differentially expressed gene are shown as connected smaller nodes and grouped classified based on corresponding gene ontologies.

To define the potential function of candidate genes identified in the GWAS pipeline, we generated microarray‐based transcriptome data from 16 days after fertilization developing seeds of RSQ lines containing extremely low or high levels of each micronutrient and mostly possessing inferior or superior functional haplotypes for each respective micronutrient to check differentially regulated genes (Figure S2; Table S6). For Mg, the results showed that two identified key candidate genes were differentially regulated: *CYP99A2* (logFC = 1.75, *P* = 0.02, β = −3.57) and LOC_Os04g10380 (logFC = 1.19, *P* = < 0.00047, β = −0.04) (Table S6.1b). Most of the high Mg‐containing lines having the superior functional haplotypes of the identified candidate genes showed upregulation of these candidate genes as compared with lines with low Mg levels (Table S6.1b). Results also showed coexpression of these candidate genes in green and blue modules with multiple genes such as OsABC transporters, non‐yellow coloring genes, grain size and number, vacuolar Fe transporter, metallothionein, auxin response factors, and other genes involved in RNA regulation, signaling, protein modification, development, and unclassified functions (Figure 6a,b; Table S7.1). The gene *CYP99A2*, which was also a candidate gene for P, showed differential expression in RSQ lines with contrasting P levels and functional haplotypes (logFC = 1.80, average expression = 6.25, *P* = 0.0025, β effect = −1.60) (Table S6.2b). Most of the lines with low P levels and inferior functional haplotype showed downregulation of *CYP99A2* (Figure S2; Table S6.2b). This gene was also coexpressed with other cytochrome P450, phosphate transporters, starch synthesis genes, receptor‐like kinases, probenazole‐inducible genes, chitinases, early‐light inducible genes, and other genes involved in response to water stress, heat shock, and pathogen attack, among others (Figure 6c; Table S7.2). For S, three candidate sulfate transporters (SULTR2;2, SULTR2;1, SULTR1;1) were also found to be differentially expressed (logFC range = −1.2 to −1.4, *P*‐value range = <0.001 to 0.003) (Table S6.3b), wherein both *SULTR2;2* and *SULTR2;1* were coexpressed in the same cyan module while *SULTR1;1* was in the yellow module. Most of the high S‐containing lines possessing superior functional haplotype exhibited downregulated expression of these candidate genes (Table S6.3b). These sulfate transporters were coexpressed with some sulfur transferases, OsABC transporters, purple acid phosphatases, serine carboxypeptidases, and other genes involved in stress response, development, transport, protein modification, signaling, and metabolism (Figure 6d,e; Table S7.3). For Cu, three key candidate genes were also differentially expressed: *OsHMA5* (LOC_Os04g46940), *OsPME15* (LOC_Os07g49010), and *OsADF6*. *OsADF6* was coexpressed in the red module and was downregulated in low Cu‐containing lines having the inferior functional haplotype (Table S6.4b). The gene *OsADF6* was coexpressed with genes involved in phytochelation, starch synthesis, cellulose synthesis, and carotenoid biosynthesis, and with other loci playing various roles in metabolism, protein modification, transport, signaling, and other unclassified functions (Figure 6f; Table S7.4). On the other hand, both *OsHMA5* and *OsPME15* were coexpressed in the cyan module and were mostly upregulated in low Cu‐containing lines (Table S6.4b). These two candidate genes were coexpressed with other genes such as OsNRAMP6, *S*‐adenosyl‐*I*‐methionine synthetase2, Fe‐S cluster protein 43, OsABC transporters, cation protein exchanger 15, aquaporin gene, heavy‐metal associated protein 25, some chitinases and other coexpressed genes involved in metabolism, protein modification, RNA regulation, photosystem light reaction, cell/cell wall/membrane processes, stress response, development, transport, and signaling (Figure 6g; Table S7.4).

### Haplotype mining within IRRI breeding lines

**Figure 7:**
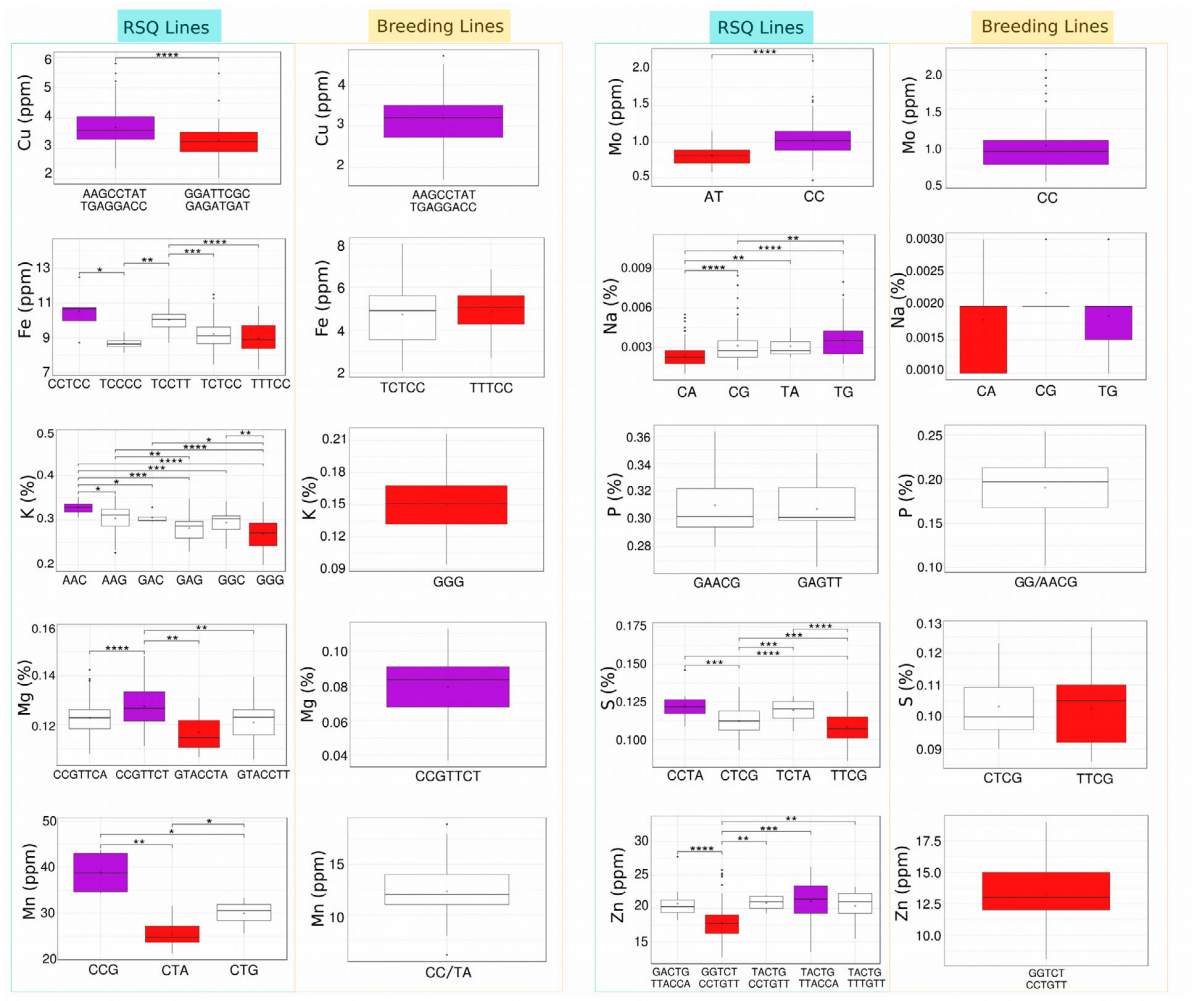
Functional haplotypes formed from common top non‐redundant single nucleotide polymorphisms of candidate genes per micronutrient mined in the RSQ panel and IRRI breeding lines showing varying levels of micronutrients. Alleles of the top candidate genes per micronutrient were mined and compared for the RSQ and breeding lines. Superior and inferior alleles per mineral comparing the two sets of lines are colored in purple and red, respectively. Statistical significance of differences is indicated by asterisks: *P < 0.05; **P < 0.01; ***P < 0.001; ****P < 0.0001.

To determine if the existing IRRI breeding lines contain advantageous alleles for high mineral content, we mined the alleles of functional haplotypes that explain higher PVE as defined from the top non‐redundant SNPs across the candidate genes in the RSQ panel and the IRRI breeding lines. In general, a smaller number of allelic groups was observed in the IRRI breeding lines as compared with the RSQ lines (Figure 7). For instance, six allelic groups were identified in the RSQ panel for K, while only one allelic group, represented by more than three samples, was identified in the IRRI breeding lines. Moreover, inferior functional haplotypes for Fe, K, S, and Zn were present mostly in the IRRI breeding lines while superior functional haplotypes were identified in the RSQ panel. For instance, the superior functional haplotype (CCTCC) showing approximately 10.5 ppm mean Fe found in the RSQ panel was absent in the IRRI breeding lines. Similarly, the superior functional haplotype with about 21 ppm mean Zn in the RSQ panel was also missing in the IRRI breeding lines. Nonetheless, the superior functional haplotypes for Cu, Mo, and Mg were identified in almost all of the IRRI breeding lines while the inferior functional haplotypes were under‐represented (Figure 7).

## DISCUSSION

### Systems genetics through GWAS unravel rare alleles for enriched micronutrients

As GWAS allows the identification of multiple genomic regions associated to the trait of interest while exploiting the genetic variation of diversity lines, it is a useful tool for exploring the polygenic nature of rice grain micronutrients (Descalsota‐Empleo et al., 2019; Norton et al., 2014; Swamy et al., 2018; Tan et al., 2020). However, as GWAS tests for a single SNP position at a time, it is considered to have a limitation in projecting the actual genetic architecture of complex traits, which are simultaneously regulated by multiple loci (Kaler & Purcell, 2019). To overcome this limitation, we have implemented the gene‐level analyses and GSA approach through MAGMA, which considers the LD of numerous SNPs and determines the joint effects of multiple candidate genes (de Leeuw et al., 2015) linked with complex traits such as rice micronutrients. The challenge in identifying significant regions in GWAS with low false discovery rate (FDR) was overcome by the implementation of different downstream approaches such as GSA and targeted associations. By further dissecting important candidate genes and implementing stringent filtration methods, we were able to narrow down to the most important candidate genes and SNPs, which explain large percentage variations for various micronutrients in the RSQ *indica* lines. The PVE values for each candidate gene per trait ranged from 5% to >20%. Considering all candidate genes per trait with a cumulative PVE value by linking high‐value multiple candidate genes per trait, genome‐wide combined multi‐haplotypes explained higher PVE ranging from more than 11% to almost 40%. While some chromosomal peaks based on GWAS results alone explained high percentage variation per mineral, the PVE of multi‐haplotypes (top SNPs from multiple candidate genes) exhibited higher PVEs in general. This result suggested that several candidate genes would be associated with the corresponding micronutrients of interest. Furthermore, the combination of the association network approach to identify key SNP variants influencing multiple micronutrients, and the genome‐wide epistatic interactions analysis of the 109 candidate loci delineated genetic interactions between multiple micronutrients. The gene regulatory networks derived for the key genetic variants identified in GWAS further complemented and validated the high value genes for mineral accumulation in brown rice. Previous studies on micronutrients in rice identified QTL or large genomic regions linked with different elements. In this study we identified high‐value target candidate genes from the stable QTLs determined by previous reports (Aung et al., 2019; Suman et al., 2021). In addition, we mined important alleles from these markers, which could be used in the future for breeding strategies towards rice biofortification.

### GWAS coupled genome‐wide epistatic interactions delineate complex regulations between multiple minerals

Epistatic interactions between genomic regions, can account for the hidden quantitative genetic variations and contribute large effects (Carlborg & Haley, 2004). We observed multiple epistatic interactions for almost all of the top non‐redundant SNPs linked with the 12 micronutrients. Multiple SNPs linked with Cu exhibited positive epistatic interactions to several SNPs linked with Zn. The multiple epistatic interactions of the SNPs reveal a portion of the complex genetic architecture of Zn and other micronutrient accumulation in rice grains. Na showed a significantly positive correlation with K. One of the candidate genes for Na was *OsHKT1;5*, which encodes for HIGH‐AFFINITY K^+^ TRANSPORTER 1;5 involved in K and Na ion homeostasis (Kobayashi et al., 2017). It exhibits positive epistatic interaction. SNPs linked with Zn were linked epistatically to multiple SNPs for other nutrients such as Mn, Na, and Cu, suggesting complex regulation among nutrients. Previous studies also reported several epistatic interactions for different micronutrients such as Cu, Mg, Fe, Na, Zn, Ca, K, and Mo, among others, explaining a wide range of phenotypic variation percentage (Descalsota‐Empleo et al., 2019; Suman et al., 2021; Swamy et al., 2018). Such epistasis implies the interactions of multiple genes resulting in differential phenotypic expression eventually contributing to variations in multi‐micronutrient accumulation. In addition, such correlations suggest that common mechanisms regulated by multiple common genes may underpin the accumulation of micronutrients in rice grains eliciting large genetic effects similar to the large‐effect QTLs per micronutrient (Carlborg & Haley, 2004; Descalsota‐Empleo et al., 2019).

### Genetic variants influencing Cu and Mg in rice grains

We identified hot‐spot QTL regions for Cu and Mg in the lower and upper arms of chromosome 4, respectively. Interestingly, most of the significant SNPs in these QTL regions have low *r* ^2^ values, although they may have moderate to high D′ values. This observation may imply the rarity of some SNPs within these regions and different combinations of alleles may be affecting the concentration of these micronutrients. In addition, only few SNPs were excluded when non‐redundant significant SNPs were identified and the SNPs within those large QTL regions in the case of Cu exhibited both negative and positive effects. Within the QTL region for Cu, the gene *COPPER TRANSPORTING ATPASE* HMA5 (*OsHMA5*) was located along with other candidate genes with high PVE values. *OsHMA5* and pectin methylesterase 15 (*OsPME15*) were upregulated in low Cu‐containing lines. *OsHMA5* and *OsPME15* were coexpressed in the same module along with 604 genes mostly involved in metabolism of different molecules (lipids, amino acids, hormones, major and minor CHO, and secondary metabolites), protein degradation or post‐translational modification. *OsHMA5* is a known transporter essential in promoting Cu translocation from xylem of roots and other organs to shoots (Deng et al., 2013). A homolog, *OsHMA4*, is known to prevent Cu accumulation in rice grains by sequestering Cu in root vacuoles (Huang et al., 2016). Similarly, *OsPME15* is involved in Cu tolerance as it may regulate pectin methyl esterification in cell walls (Krzesłowska, 2011). Within the same module, several ABC transporter genes along with some heavy metal‐associated genes were also coexpressed. One strongly coexpressed gene, *OsCAO2*, encodes for a chlorophyllide A oxygenase, which is known to bind Fe and S molecules and is involved in tetrapyrrole synthesis (Lee et al., 2005). Tetrapyrroles are macrocyclic molecules known to be capable of absorbing visible light or accepting different redox states (Brzezowski et al., 2015).

A significant positive correlation was found between Cu and Zn in previous reports (Jiang et al., 2007; Li et al., 2015). Although no common strong candidate genes were identified for these two micronutrients, the genes for Cu linked SNPs (LOC_Os04g46600, *SPL7*, *TIP5;1*, *D17*, *UGT93B*, and *CYC2;1*, among others) were epistatic to several Zn‐linked SNPs. Some of the top candidate genes for Cu encode proteins with Zn components such as LOC_Os04g46920 and LOC_Os04g46600, which encode for Zn knuckle domain‐containing protein and Zn finger protein, respectively. Interestingly, a central P‐linked SNP (snp_04_5518113) of LOC_Os04g10200 exhibited negative epistatic interactions with Cu‐linked SNPs. Antagonistic effects of P with Zn and Cu were reported previously (Ova et al., 2015; Wang et al., 2012). A decrease in Cu concentration (but not Fe) was recently reported in maize grains upon P fertilizer application (Zhang et al., 2020). Accounting for 65%–85% of total seed P, the principal storage form of P in cereal grains is in the form of phytic acid (PA) (Raboy, 2000), which is negatively charged thereby able to chelate cations strongly such as Fe, Zn, and Cu, as well as K, Mg, and Ca among others (Raboy, 2003). Chelation forms highly insoluble salts that prevent important nutrients from being absorbed by the human intestine, leading to micronutrient deficiency (Mitchikpe et al., 2008; Perera et al., 2018). Rice seeds contains approximately 70% PA of the total P in seeds, 80% or more of the PA is found in the aleurone and pericarp layers (Iwai et al., 2012). It has also been shown that many genes linked with Pi (inorganic phosphate) are activated with Zn deficiency (van de Mortel et al., 2006), while genes linked with Fe and Zn are upregulated with Pi deficiency (Bustos et al., 2010). These associations among Zn, Cu, P, and Fe should be further explored through more in‐depth studies.

For Mg, different candidate genes encoding momilactone synthases, amidases, cytochrome P450, and subtilisin among others were found. For lines with contrasting Mg, two candidate genes CYP99A2 (LOC_Os04g10160) and LOC_Os04g10380 were identified as critical. The LOC_Os04g10380 encodes for a glycine‐rich protein with unknown function and is found coexpressed with several genes encoding ABC transporters, protein phosphatases, and auxin response factors. CYP99A2 encodes for a cytochrome P450 99A2, which has a metal binding site and is involved in momilactone phytoalexins biosynthesis for defense response (Shimura et al., 2007). The CYP99A2 was coexpressed with few genes such as non‐yellow coloring genes, vacuolar Fe transporter 2, plasma membrane H^+^‐ATPase 7, and ent‐kaurene synthase 7, which has Mg ion binding gene ontology. Similarly, CYP99A2, which was also a candidate gene for P, was also differentially expressed in lines with contrasting P accumulation. Lines with low P levels showed downregulated CYP99A2. This gene has been coexpressed with different genes encoding chitinases, actin depolymerizing factor 3, receptor‐like cytoplasmic kinases, blast resistance 7b, phosphate transporter, ABC transporter, starch synthesis genes, early light‐inducible protein, and pathogenesis linked genes among others.

Notably, Mg had a strong positive correlation with P and shared a common candidate gene *CYP99A2* encoding the cytochrome P450 protein, which is involved in momilactone phytoalexins biosynthesis for plant defense (Shimura et al., 2007). Based on GSA, three candidate genes in the top gene set for P were also linked with Mg, based on GWAS. Mg also showed significant positive correlation with Fe. While *CYP99A2* was not a strong candidate for Fe, this protein has an Fe ion‐binding capacity based on database mining. Interestingly, Mg was negatively correlated with chalkiness and positively correlated with protein content, highlighting its importance in protein synthesis and transport of photo‐assimilates (Tränkner et al., 2018). In addition, a previous study also observed chalkiness reduction and hardness increase for rice varieties able to accumulate Mg, K, and P in their grains (Zohoun et al., 2018).

### Genetic variants influencing S in rice grains

The candidate genes for S with PVE >10% each such as *OsSultr1;1*, *OsSultr1;2*, *OsSultr2;1*, and *OsSultr2;2* encoding for sulfate transporters were consistently identified in all our approaches, suggesting their prime relevance in controlling S concentrations for brown rice. Three candidate genes (*SULTR2;2*, *SULTR2;1*, and *SULTR1;1*, which all encode for sulfate transporters) for sulfur were also differentially expressed in lines with contrasting S levels. *SULTR2;2* and *SULTR2;1* were coexpressed with multiple genes such as ABC transporters, receptor‐like cytoplasmic kinases, calcium sensors, phytochelatin, sulfotransferase, disease resistance, sweet genes, high‐affinity K^+^ transporter18, chitinase, carotene hydroxylase 1, stress‐related genes, laccase, and other genes. Similarly, *SULTR1;1* was coexpressed with different genes encoding thionin, ferulate 5‐hydroxylase 1, sulfur transferase, ABCG45, TIP1;1, and purple acid phosphatase 10c, among others. Most of the high S‐containing lines have more downregulated expression of these genes. *OsSultr1;1* has been known to provide tolerance to abiotic stress under low S conditions (Kumar et al., 2019). Similarly, *OsSultr2;1* and *OsSultr2;2* are known to facilitate sulfate distribution to the leaves under limited S environments (Takahashi, 2010). Sulfur showed significantly positive correlation with multiple micronutrients such as Fe, Mg, K, P, and Mo, which in turn were also positively correlated with almost one another. Also, P and S showed positive correlation with multiple micronutrients such as Mg, Fe, and Mo, which also positively correlated with protein content. Interestingly, two upstream SNPs from sulfate transporter genes associated with S showed negative epistatic interactions with multiple SNPs linked to Cu. A previous report showed that Cu(I) when complexed with thiol‐S ligands found in root epidermis and xylem showed decreased Cu levels in rice (Cui et al., 2019).

### Genetic variants influencing Fe and Zn in rice grains

Biofortification of rice is largely focused on Fe and Zn enrichment. Deficiencies in these two micronutrients largely account for the hidden hunger, particularly in developing countries. Our results depicted significant positive correlation between Zn and Fe suggested common metabolic regulators and feasible concurrent biofortification for both ions (Calayugan et al., 2020; Maganti et al., 2020; Xu et al., 2015). Descalsota‐Empleo et al. (2019) performed GWAS using 152 colored rice accessions with 22 112 SNPs to identify QTLs linked with Fe, Zn, and other agronomic traits and they identified three QTLs for Fe and Zn, which co‐located with metal homeostasis genes such as *OsCNGC16*, *OsDof*, *OsHMA9*, *OsNRAMP3*, *OsbZip85*, *OsNRAMP7*, and *ZFP252* (Descalsota‐Empleo et al., 2019). Other known candidate genes identified for Fe and/or Zn include *OsNAS3*, which leads to an increase in Zn concentration as well as Fe and Cu when activated (Lee et al., 2009), and *OsZIP5*, which is known to transport Zn from the soil and increase Zn concentration in roots (but decrease Zn in shoots) upon overexpression (Lee et al., 2010). One NAS ortholog, *OsNAS2*, was previously validated by Trijatmiko et al. (2016) contributing to higher Fe and Zn concentrations when overexpressed. We found three common candidate genes within 59.61 kb with very high PVEs for both Fe and Zn: *OsLonP3*, *OsDLN197/TOPBP1B*, and LOC_Os07g49020 (expressed protein). *OsNAS3* was also a common candidate gene for both Fe and Zn but with a slightly lower PVE for Fe (7.28%) compared with Zn (15.35%). Most of the candidates for Zn and Fe were in a large QTL (approximately 164 kb) on chromosome 7 covering the four common candidate genes known within this region suggesting complex regulation of this QTL. These common candidate genes for Fe and Zn could be found near or overlapping with QTLs previously identified in multiple locations for brown rice or polished rice (Aung et al., 2019; Suman et al., 2021). Such information further validates the outcomes of this study, which homes in to the potential target genes. Considering the functional haplotypes formed through combining top non‐redundant SNPs from the common candidate genes for Fe and Zn, including *OsNAS3* and *OsZIP5*, we found 14 samples possessing the superior functional haplotype CCTGT, which showed 10.57 ppm mean Fe and 20.32 ppm mean Zn.

Validation of these markers using a large collection of donor lines, specific biparental mapping studies, and QTL pyramiding are recommended strategies to validate the importance of these markers (Swamy et al., 2016) to increase simultaneously the Zn, Fe, and other nutrients. Although Fe, Zn, Mn, and Cu mostly accumulate in the grain aleurone layer where PA chelates these minerals to form phytate (Cichy & Raboy, 2009), other studies show that Zn is bound more to the proteins while Fe mostly associates with PA (Kutman et al., 2010). It has also been pointed out that a high correlation of Zn and Fe was usually observed in brown rice but not in polished rice (Sala et al., 2013; Swamy et al., 2016). Hence, although Fe and Zn showed co‐localization of associated QTL/genes, more in‐depth studies need to be conducted to understand the relationship of these two micronutrients at the molecular level.

The RSQ diversity lines possessed four sets of superior alleles compared with the inferior alleles of the breeding lines and the Zn levels were approximately 20 and 13 ppm mean Zn respectively. The recommended dietary intake for Zn ranges from 8 to 11 mg day^−1^. Hence, in a regular cup of rice (200 g), diversity lines with the superior alleles can contain approximately 6.66 mg of Zn and this could fulfill the recommended dietary allowance of 8–11 mg day^−1^ in male and female adults within two meals given at most one cup of rice per meal. Likewise, diversity lines possessed superior alleles such as CCTCC and TCCTT showing 10 ppm to 10.5 ppm mean Fe content compared with some breeding lines, which possessed the inferior allele TTTCC with less than 6 ppm mean Fe content. Hence, in a regular cup of rice (200 g), diversity lines with the superior alleles can contain approximately 3.33 mg of Fe and this could fulfill the recommended dietary allowance of 8–18 mg day^−1^ in male and female adults within two to three meals given at least one big cup of rice (300 g) per meal. In contrast, some breeding lines (*n* = 28) possessing the inferior allele TTTCC may only contribute 1.2 mg Fe per cup, hence requiring a higher quantity of rice for consumption. The lines possessing the superior alleles for Fe also showed >20 ppm mean Zn content, suggesting these lines could be used as donor lines for simultaneous Fe and Zn future biofortification strategies.

Aside from Zn and Fe, this approach might also be applicable with other important rice grain micronutrients identified in the present study to eliminate mineral deficiencies globally. In this study, several RSQ *indica* donor lines from the diversity panel with superior alleles were identified and may be used as potential donors for enriching mineral content in rice grains in future breeding programs while ensuring higher yield and other important traits of interest.

## EXPERIMENTAL PROCEDURES

### Plant materials and growth conditions

A diverse set of 318 *indica* accessions identified from the 3000 Rice Genomes Project (Wang et al., 2018) were grown at the Zeigler Field Experimental Station, International Rice Research Institute (IRRI), Laguna, Philippines (14°10 0 N, 121°15 0 E) in a completely randomized block design with three replications during the 2015 dry season, employing standard crop management practices (Misra et al., 2017). Manual harvesting was performed and grains were threshed, oven‐dried, and stored in a room at a temperature of 18°C.

### Ion measurement

Preparation and analysis of samples were conducted following the methods of Molina et al. (2019). From each cultivar with four replicates, 0.600–0.625 g of rice grain samples were placed in a culture tube (25 × 200mm) and washed with 12 ml of 1:10 of HClO_4_:HNO_3_ mixed acid solution. The samples were then pre‐digested starting at 60°C for 25 min and then digested following the digestion temperature scheme until a colorless or slightly yellow clear digest remain (Molina et al., 2019). Upon cooling down of the digest, 20 ml of 1% HNO_3_ was added, and then the tubes were warmed for 35 sec using a digestor and mixed using a vortex mixer. The samples were finally diluted to the 25‐ml mark with 1% HNO_3_, mixed using a vortex mixer, and transferred into polypropylene tubes. The digests were then analyzed for concentrations of 12 micronutrients (Al, Ca, Cu, Fe, K, Mg, Mo, Mn, Na, P, S, Zn) for 318 diverse *indica* lines grown in 2015 dry season at the experimental field of IRRI were determined for brown rice samples using inductively coupled plasma optical emission spectrometry as discussed in detail by Molina et al. (2019). In addition, four biofortified lines and 92 IRRI breeding lines were also phenotyped for the aforementioned micronutrients except for Ca and grain quality traits. Principal components analysis was performed using the *prcomp* function in R (R Core Team, 2021) for the RSQ and biofortified lines only, while the IRRI breeding lines were used for haplotype mining analyses. Correlation analysis was also performed for the micronutrients and grain quality traits of the *indica* diversity panel using the Pearson correlation coefficient (Pearson, 1895) in R.

### GWAS

Genotype data were filtered using PLINK 1.9 (Chang et al., 2015) with the following criteria: 1% missingness rate per marker, 5% missingness rate per sample, and 10% minor allele frequency. After filtering the biallelic 2 863 830 SNPs, 1 024 492 SNPs remained with 303 RSQ samples, except for Al wherein 301 samples were retained with 1 029 071 SNPs. Phenotype values were transformed using WarpedLMM (Fusi et al., 2014), which incorporated the genotype profiles of the samples during transformation. The Balding–Nichols kinship matrix was calculated using the EMMAX‐kin function (Kang et al., 2010) and used to correct for cryptic relatedness. GWAS was performed using EMMAX (Kang et al., 2010). Manhattan and quantile‐quantile plots were created using R. The Bonferroni threshold was set to 0.05/*s* where *s* is the total number of filtered SNPs used in GWAS. The suggestive line was set to *P* < 0.00001 similar with previous studies (e.g., Chen et al., 2014; Misra et al., 2018; Wang et al., 2015). The FDR was also calculated using the Benjamini–Hochberg method (Benjamini & Hochberg, 1995). Broad sense and narrow sense heritability values were calculated using the heritability package (Kruijer et al., 2015) in R. The PVE by each peak found in single‐locus GWAS was calculated using LDAK (Speed et al., 2020).

### Gene‐level analyses and GSA

Gene‐level analyses were performed for each of the 12 micronutrients using MAGMA (de Leeuw et al., 2015) to calculate the joint association of all the SNPs located in a given gene based on the *P*‐values obtained by single locus GWAS using the EMMAX method. The top 20 genes with the lowest *P*‐values based on gene‐level analysis per micronutrient were used to test the joint association of different combinations of candidate genes to each corresponding mineral. Several sets of genes were produced by deleting one gene from the set of the top 10 genes based on the *P*‐value for each gene set after computing correlations of neighboring genes and other gene‐level metrics such *Z*‐score and *P*‐value per gene. The minimum number of gene set with the highest β with the FDR <0.05 was considered as the top gene set for each micronutrient. The candidate genes included in the top gene set were then subjected to targeted association analysis done per micronutrient as well.

### Targeted association analysis

Targeted association analyses were performed using EMMAX (Kang et al., 2010) for all candidate genes found in single‐locus GWAS and top1 gene set, and known genes based on literature review and database mining. Significant SNPs per candidate gene were filtered based on Bonferroni threshold per candidate gene. The top candidate genes per mineral were mapped to corresponding chromosome positions using CViT (chromosome visualization tool) (Cannon & Cannon, 2011). The LD plots of tag SNPs for the micronutrients were created using Haploview (Barrett et al., 2005) based on the confidence interval scheme (Gabriel et al., 2002). The strength of recombination was presented in colors based on 95% confidence intervals on D prime (dark gray: strong LD, gray: inconclusive, white: strong recombination) while *r* ^2^ values for each SNP pair were represented by color‐coded circles. The significance of each tag SNP was also shown in barplots. The thickness of the bars represents the absolute β value of each tag SNP (thicker bar suggests higher absolute β), while the black and red colors of the bars indicate positive or negative β, respectively.

### Narrowing down of candidate genes and association networks

Significant SNPs per candidate gene were pruned (*r* ^2^ < 0.2, 75 kb window) and used to calculate PVE using the *reml* function in LDAK (Speed et al., 2020). For candidate genes found in large QTL regions, the SNPs were thinned out using the heritability model in LDAK with the threshold *r* ^2^ < 0.98 within 75 kb window. The top candidate genes were further filtered based on the PVE range of candidate genes per micronutrient with the following thresholds: PVE >8% was used for Fe and Mn, >5% for Al, and >10% for Cu, S, Na, Mg, P, Zn, Ca, and K. Association networks of candidate genes per micronutrient based on filtered significant SNPs were created using cytoscape (Shannon et al., 2003). The candidate genes were used as the target nodes connected to the trait of interest treated as the source node. The thickness of the edges/connectors are based on the PVE per candidate gene. Then, the total PVE for all candidate genes per micronutrient based on the top non‐redundant SNPs was calculated. Alleles for the significant non‐redundant SNPs of the filtered top candidate genes were mined across the RSQ panel and IRRI breeding lines. The allelic groups per panel were then compared using *t*‐test or Wilcoxon test with adjusted *P*‐values based on Holm's method (Holm, 1979), and boxplots were constructed using R (R Core Team, 2021).

### Epistatic interactions analysis

In total, 103 unique significant SNPs linked with the 12 micronutrients after PVE filtration were subjected to epistatic interaction analysis (Misra et al., 2021) against each micronutrient trait already defined based on GWAS, GSA, and targeted association analyses. Pairwise interactions were considered significant when *P* < 0.001. WarlpedLMM (Fusi et al., 2014) transformed normally distributed phenotype data along with genotype data was used as input in the linear regression model for epistatic interaction. Epistatic interaction was carried out using PLINK 1.9 (Chang et al., 2015). Significant pairwise interactions plotted using cytoscape (Shannon et al., 2003) where each SNP showed ≥2 interaction. Red colored edges represent a positive β value, whereas blue colored edges are the negative β values.

### Gene regulatory networks derived from the microarray transcriptome data

Developing seeds at 16 days after fertilization were obtained from 195 RSQ *indica* lines for single channel microarray analysis. A GCRMA‐based normalization of the microarray data was then performed using the justGCRMA function (Wu et al., 2019) implemented in R software. Lines with contrasting micronutrients based on the combinations of alleles from top candidate genes were subjected to further analysis. The WGCNA package (Langfelder & Horvath, 2008) implemented in R software was used to identify clusters of correlated genes. Using the network construction algorithm (Zhang & Horvath, 2005), first the correlation matrix (coefficient <0.75) was transformed into an adjacency matrix, which was then converted into a topological overlap matrix. Subsequently, hierarchical clustering was performed on topological overlap similarity using cutreeDynamic function to identify clusters (Zhang & Horvath, 2005). Eigenvectors for each cluster were calculated and those having a similar eigenvalue (threshold 0.25) were merged using the merge Close Modules function (Zhang & Horvath, 2005). Nodes having an adjacency value of 0 were removed. Each module per micronutrient was searched for any candidate genes. cytoscape software was used to visualize the final coexpression network: genes were represented as nodes and connection strengths as weighted edges (>0.1, except for SULTR2;1/SULTR2;2 network where edge weight was >0.03). The gene names for each loci were searched in the Oryzabase (Kurata & Yamazaki, 2006).

## AUTHOR CONTRIBUTIONS

EAP conducted most of the analyses and interpretation and wrote the manuscript; GM was involved in the development of the analytical pipeline and performed epistasis analysis; NS conceptualized the research, supervised all analyses. NS and AK critically reviewed/edited the manuscript. All authors contributed to the revisions of the manuscript.

## CONFLICT OF INTEREST

We declare no conflict of interest regarding this manuscript.

## Supporting information


**Figure S1.** Correlation of micronutrients and some grain quality traits of the *O. sativa* subsp. *indica* RSQ lines (colored boxes are significant at *α* = 0.05, with purple and green shades showing negative and positive correlations, respectively).

**Figure S2.** Hierarchical clustering of contrasting *O. sativa* subsp. *indica* RSQ lines per mineral based on microarray data.

**Figure S3.** Pipeline used for identifying and narrowing down the candidate genes linked with the 12 micronutrients of a diverse panel of *O. sativa* subsp. *indica* RSQ lines.

**Table S1.** Significant SNPs passing the acceptable significance threshold *P* < 0.00001 from GWAS of each mineral of *O. sativa* subsp*. indica* RSQ lines.

**Table S2.** Top set of candidate genes jointly associated to each mineral of the *O. sativa* subsp*. indica* RSQ lines identified through gene set analysis using MAGMA.

**Table S3.** Results of targeted association on the candidate genes for 12 micronutrients of the *O. sativa* subsp*. indica* RSQ lines.

**Table S4.** Summary of top candidate genes for 12 micronutrients from the *O. sativa* subsp*. indica* RSQ lines after PVE filtration.

**Table S5.** Significant pairwise epistatic interactions (*P* < 0.001) of top‐nonredundant SNPs linked with multiple minerals in brown rice (*O. sativa* subsp*. indica*).

**Table S6.** Contrasting lines used for calculating DEGs for each micronutrient and list of differentially expressed genes between superior and inferior RSQ lines in terms of Mg levels at log fold + −1 and *P* value = <0.05 along with their annotation and normalized value.

**Table S7.** Coexpressed genes of some differentially expressed candidate genes based on microarray analysis of contrasting *O. sativa* subsp*. indica* RSQ lines.

## Data Availability

All relevant data can be found within the manuscript and its supporting materials.
