# Supplementary material for: Unraveling the genetics underlying micronutrient signatures of diversity panel present in brown rice through genome–ionome linkages
Source: Plant J. 2023 Jan 18;113(4):749–71. doi: 10.1111/tpj.16080 (PMC10952705; doi:10.1111/tpj.16080)
Supplement: Supplementary file 1 — Figure S1. Correlation of micronutrients and some grain quality traits of the O. sativa subsp. indica RSQ lines (colored boxes are significant at α = 0.05, with purple and green shades showing negative and positive correlations, respectively). [file TPJ-113-749-s001.pdf]

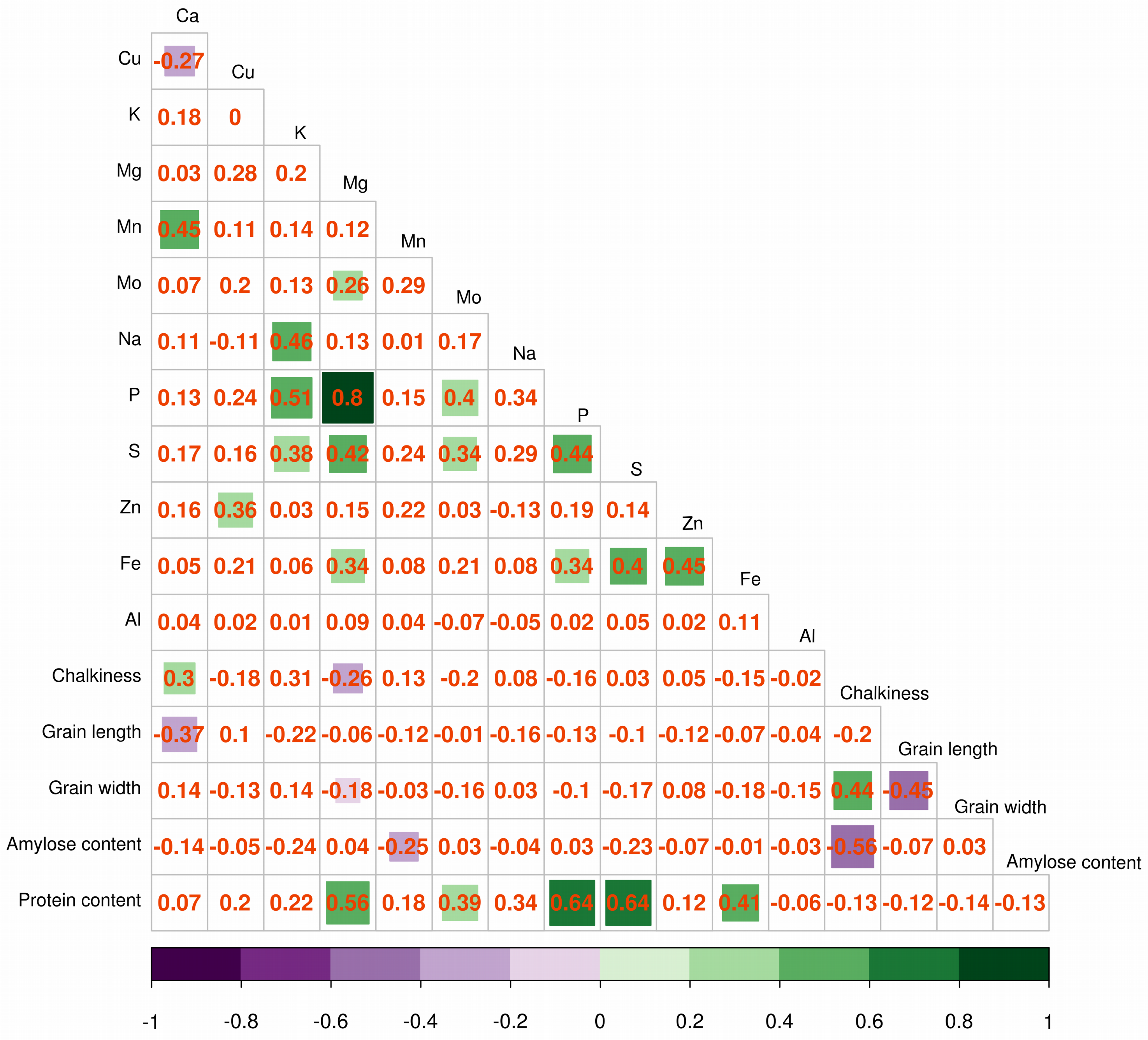

Figure S1. Correlation of micronutrients and some grain quality traits of the *O. sativa* subsp. *indica* RSQ lines (colored boxes are significant at α=0.05, with purple and green shades showing negative and positive correlations, respectively).
