# Supplementary material for: Unraveling the genetics underlying micronutrient signatures of diversity panel present in brown rice through genome–ionome linkages
Source: Plant J. 2023 Jan 18;113(4):749–71. doi: 10.1111/tpj.16080 (PMC10952705; doi:10.1111/tpj.16080)
Supplement: Supplementary file 2 — Figure S2. Hierarchical clustering of contrasting O. sativa subsp. indica RSQ lines per mineral based on microarray data. [file TPJ-113-749-s010.pdf]

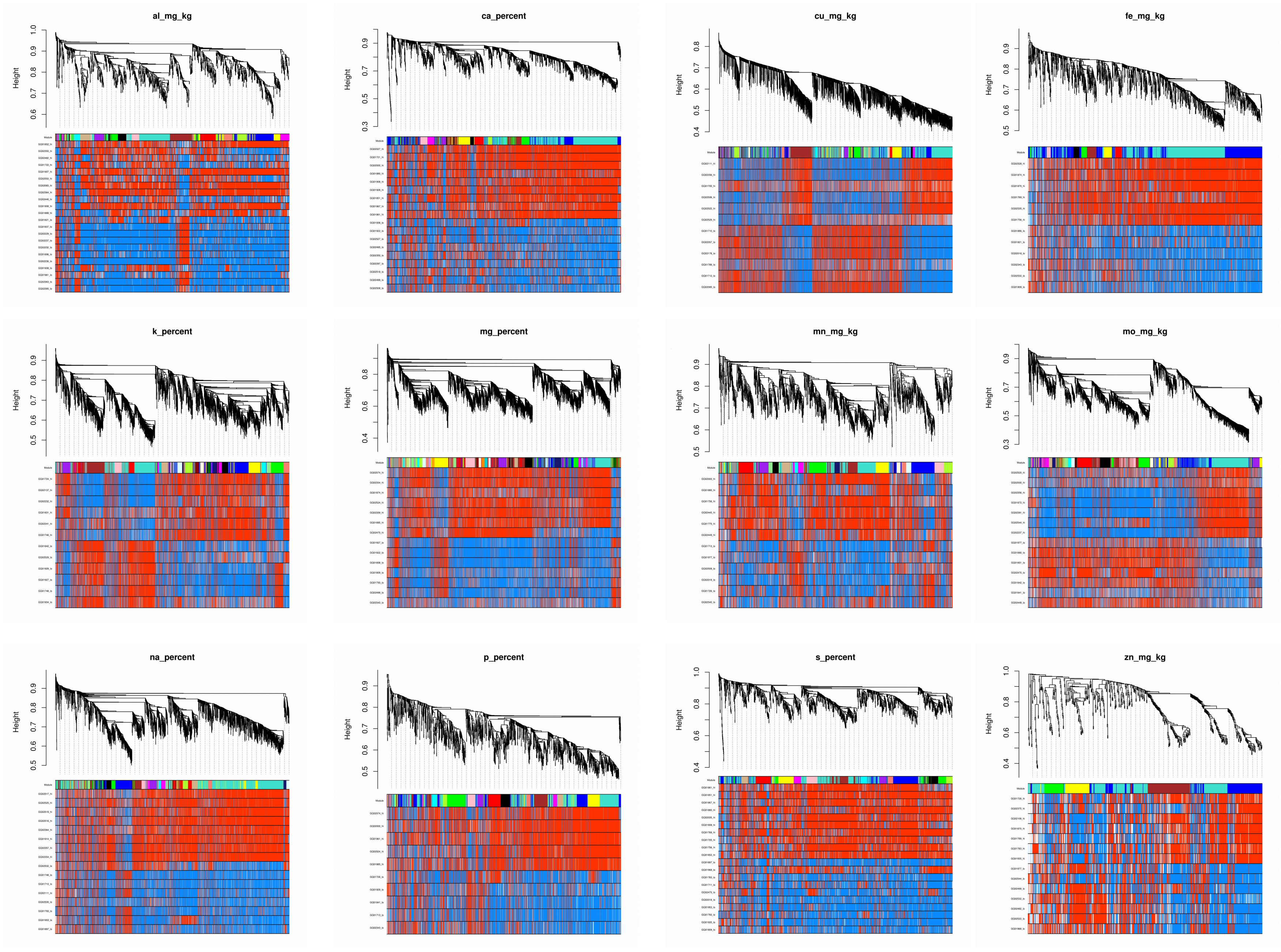

Figure S2. Hierarchical clustering of contrasting *O. sativa* subsp. *indica* RSQ lines per mineral based on microarray data.
