# Supplementary material for: Unraveling the genetics underlying micronutrient signatures of diversity panel present in brown rice through genome–ionome linkages
Source: Plant J. 2023 Jan 18;113(4):749–71. doi: 10.1111/tpj.16080 (PMC10952705; doi:10.1111/tpj.16080)
Supplement: Supplementary file 3 — Figure S3. Pipeline used for identifying and narrowing down the candidate genes linked with the 12 micronutrients of a diverse panel of O. sativa subsp. indica RSQ lines. [file TPJ-113-749-s008.pdf]

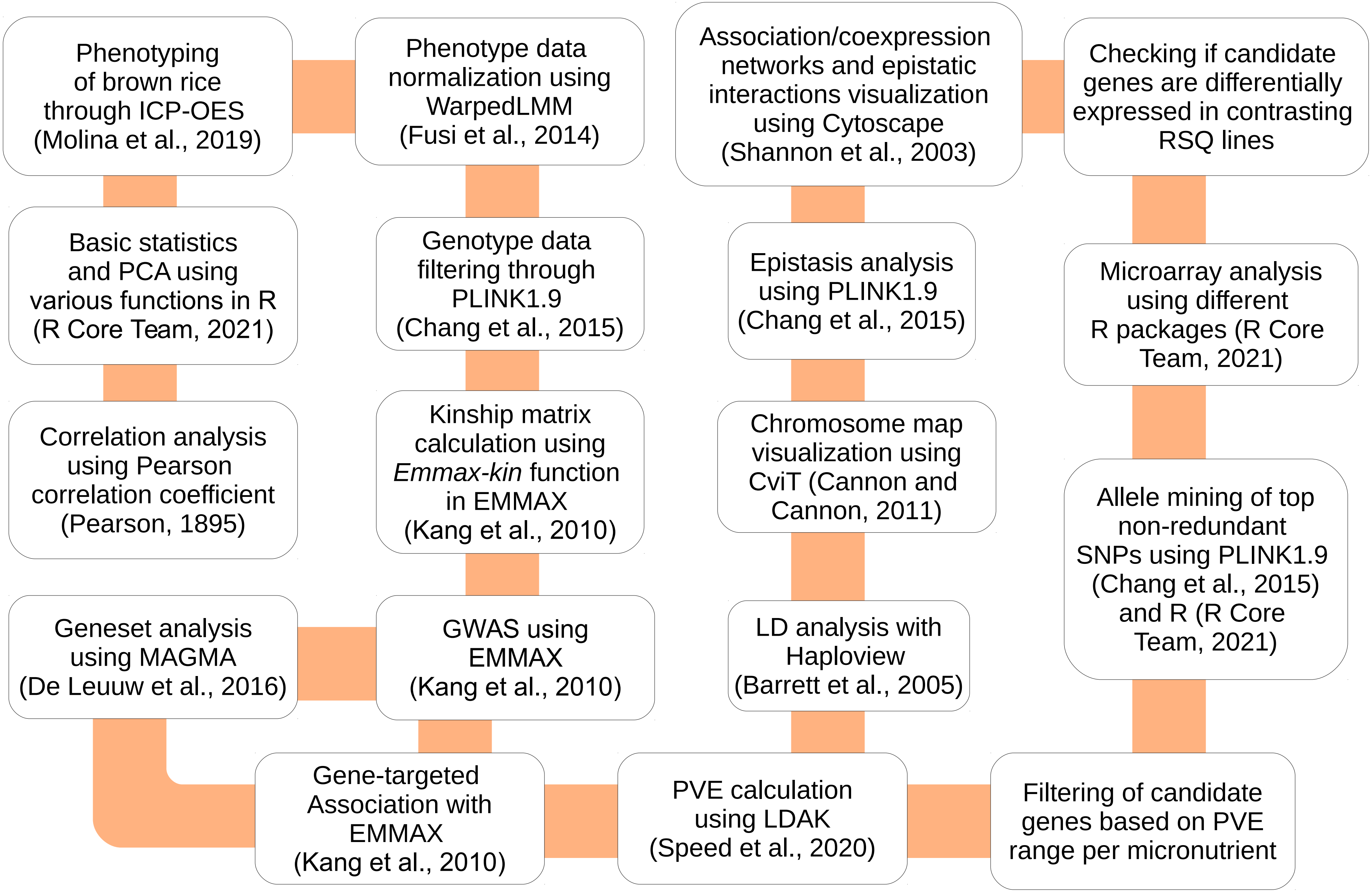

Figure S3. Pipeline for identifying and narrowing down the candidate genes linked with the 12 micronutrients of a diverse panel of *O. sativa* subsp. *indica* RSQ lines.
